# An Integrative Model of Patient-Centeredness – A Systematic Review and Concept Analysis

**DOI:** 10.1371/journal.pone.0107828

**Published:** 2014-09-17

**Authors:** Isabelle Scholl, Jördis M. Zill, Martin Härter, Jörg Dirmaier

**Affiliations:** Department of Medical Psychology, University Medical Center Hamburg-Eppendorf, Hamburg, Germany; Providence VA Medical Center and Brown University, United States of America

## Abstract

**Background:**

Existing models of patient-centeredness reveal a lack of conceptual clarity. This results in a heterogeneous use of the term, unclear measurement dimensions, inconsistent results regarding the effectiveness of patient-centered interventions, and finally in difficulties in implementing patient-centered care. The aim of this systematic review was to identify the different dimensions of patient-centeredness described in the literature and to propose an integrative model of patient-centeredness based on these results.

**Methods:**

Protocol driven search in five databases, combined with a comprehensive secondary search strategy. All articles that include a definition of patient-centeredness were eligible for inclusion in the review and subject to subsequent content analysis. Two researchers independently first screened titles and abstracts, then assessed full texts for eligibility. In each article the given definition of patient-centeredness was coded independently by two researchers. We discussed codes within the research team and condensed them into an integrative model of patient-centeredness.

**Results:**

4707 records were identified through primary and secondary search, of which 706 were retained after screening of titles and abstracts. 417 articles (59%) contained a definition of patient-centeredness and were coded. 15 dimensions of patient-centeredness were identified: essential characteristics of clinician, clinician-patient relationship, clinician-patient communication, patient as unique person, biopsychosocial perspective, patient information, patient involvement in care, involvement of family and friends, patient empowerment, physical support, emotional support, integration of medical and non-medical care, teamwork and teambuilding, access to care, coordination and continuity of care. In the resulting integrative model the dimensions were mapped onto different levels of care.

**Conclusions:**

The proposed integrative model of patient-centeredness allows different stakeholders to speak the same language. It provides a foundation for creating better measures and interventions. It can also be used to inform the development of clinical guidance documents and health policy directives, and through this support the shift towards patient-centered health care.

## Introduction

In recent years, patient-centeredness has gained in importance [1], [2], including policy and practice developments to promote patient-centered care on the level of legislation and regulation of health care [3]. In the UK, patient-centered care has been encouraged by several policy papers, e.g., the “Public and patient experience and engagement (PPE)” [4], and the “Liberating the NHS: No decision about me, without me” [5] both by the Department of Health. However, a recent BMJ editorial pointed out that part of the political discussions on “putting the patients first” seem to be “rhetorical lip service” [6]. Furthermore, the topic is on the agenda of influential British think tanks, e.g. the Health Foundation [7], and the King's Fund [8]. In the US, the Institute of Medicine (IOM) claimed patient-centeredness to be one of the six aims for improvement of the US health care system [9], [10]. The importance of patient-centered care has also been stressed by the 2010 Patient Protection and Affordable Care Act [11], which led to the formation of the Patient-Centered Outcomes Research Institute (PCORI) [12]. In Australia, patient-centeredness is one of the three core principles of the Australian Safety and Quality Framework for Health Care, which was endorsed in 2010 [13]. In Germany, the Federal Ministry of Education and Research, together with the pension and health insurance schemes, established a large research priority program on patient-centeredness and chronic diseases [14]. On the international stage, the topic has also been driven forward by various associations, e.g. by the International College of Person-centered Medicine (ICPCM), which emerged from the Geneva Conferences on Person-centered Medicine [15], [16].

According to the systematic review of Rathert et al. [17] studies on processes and outcomes of patient-centered care show generally positive relationships between patient-centered care with intermediate and distal outcomes. Yet, two Cochrane reviews [2], [18] concluded that the evidence on the effects of patient-centered interventions on patient healthcare behaviors or health status is mixed. Inconsistent results have also been described elsewhere [19], [20].

In light of this extensive amount of ongoing research on patient-centeredness and its prominent position on the political agenda, one could assume that the theoretical conceptualization of what constitutes patient-centeredness is clear. However, the literature draws a different picture, describing the concept as fuzzy [21], elusive [22], or even as a “poorly conceptualized phenomenon” [23]. Looking closely at existing conceptual work reveals that several models exist, which describe various dimensions of patient-centeredness. For example while Mead and Bower [24], [25] include five key dimensions of patient-centeredness in their model (e.g., biopsychosocial perspective, sharing power, and responsibility), Stewart et al. [26], [27] describe six elements of the patient-centered method (e.g., exploring both the disease and the illness experience, enhancing the patient-doctor relationship) and Ouwens et al. [28] include eight domains of patient-centered care (e.g., access to care, communication, and respect). Another model [29] focuses on the aspect of communication and highlights four domains of patient-centered communication (e.g., eliciting and understanding the patient's perspective). Furthermore, patient-centered care has often been described by statements of what is was not, rather than explanations of what is was, e.g. doctor-centered, disease-centered [16].

All in all, the existing literature reveals that there is little consensus on the concept's meaning. This leads to a heterogeneous use of the term [21], resulting in a wide variation in the dimensions included in scales that purport to measure patient-centeredness [30]–[32]. Thus, research results regarding the effectiveness of patient-centered interventions, found by such various measurement instruments, are inconsistent [19], [20]. Part of the mixed results regarding outcomes of patient-centered care could be explained by the variation in the definition of the concept which may constitute a barrier to the implementation of patient-centered care into routine clinical practice [33]. Recently, efforts have been made to disentangle conceptual ambiguities by focusing on specific aspects (e.g. patient-centered communication [34] or ethical considerations [35]) or disease-specific dimensions (e.g. cancer care [32]). However, a comprehensive and systematic analysis of existing conceptual definitions is lacking.

Thus, the aim of this systematic review was to identify and analyze the different dimensions of patient-centeredness described in the literature and to propose an integrative model of patient-centeredness based on these results.

## Methods

This study is part of a larger research project on the “Evaluation of dimensions and measurement scales in patient-centeredness”. More information on the project can be found in the published study protocol [36]. A registration of this systematic review in the International Prospective Register of Systematic Reviews (PROSPERO) was attempted but not possible because a review of definitions does not fulfill the inclusion criteria.

### Search strategy

The search strategy included an electronic protocol driven search, combined with a comprehensive secondary search, based on the recommendations of Greenhalgh and Peacock [37]. Original articles as well as theoretical and conceptual articles, book chapters, and books were considered. Five databases (MEDLINE, EMBASE, PsycInfo, Cochrane Library, Psyndex) were searched from their inception to January 2012. We searched for references that had the term “patient-centered” or “patient-centeredness” (both with American and British English spelling) in their title in order to restrict the work to a manageable amount of full texts, which clearly focus on the topic of interest. We did not include search terms, which are sometimes used as synonyms, e.g. “patient-focused” or “person-centered”. This restriction was necessary for reasons of feasibility. We limited the search to references published in English or German. Electronic search strategies were tailored to each database. The search strategies we used in the different databases are available in Appendix S1. The secondary search consisted of citation and reference tracking of key models of patient-centeredness. [1], [24], [26], [29], [38], [39] Furthermore, references were retrieved through personal knowledge and by contacting international experts in the field.

### Eligibility criteria, study selection and assessment of quality

Search results were imported into Endnote (Thomson Reuters, New York, USA) and duplicates removed. Publications, which had a conceptual definition of patient-centeredness or patient-centered care were eligible for inclusion in the review. We considered a definition to be conceptual if it specified “what needs to be assessed in empirical evidence” (p.157). [40]

First, two authors (JD and IS) screened titles and abstracts independently to exclude records that were clearly not relevant. Furthermore, we excluded records that were only short commentaries, conference abstracts, book reviews, letters to editors, etc. in order to keep the amount of full texts manageable. For all records that were selected by at least one of the reviewers during initial screening full text articles were retrieved. Subsequently, four team members (JZ and IS and two team members that are no co-authors) independently assessed full texts to check whether they fulfilled the inclusion criterion, i.e., included a conceptual definition of patient-centeredness in the full text. Disagreements were resolved by discussion with a third team member. We conducted no further assessment of validity or quality of the full texts as the aim was to identify a broad range of conceptual definitions used in the literature on patient-centeredness. Considering the fuzziness of definitions described earlier, we felt it would be arbitrary to rate the quality of some definitions higher than others.

### Content analysis

All articles that included a conceptual definition of patient-centeredness were included in the review and subject to subsequent conventional content analysis, in order to develop codes that are grounded in the actual data (i.e. in the identified definitions) [41]. For this purpose we identified each definition and divided it into meaningful units that we coded subsequently (for an example of coding of one definition [42] see Table 1). The coding sheet was developed in an iterative process. First, one author (JZ) randomly selected 50 full texts and initially coded the included definitions to develop a preliminary coding sheet. This sheet was then revised by discussion with JD and IS. In a next step, we coded the definitions in the remaining full texts, while continuously extending the coding sheet if new codes emerged during the analysis of new full texts. In each article the given definition of patient-centeredness was coded independently by two members of the research team (IS and JZ). Discrepancies that emerged from this multiple coding strategy gave valuable insights for refining the coding scheme and were resolved by discussion [43]. Finally we discussed the codes within the research team and grouped them into meaningful clusters [41], i.e. aggregated them into different dimensions of patient-centeredness. Content analysis was supported by the MAXQDA software (VERBI GmbH, Berlin, Germany).

**Table 1:** Coding example.

Patient centredness encompasses multiple aspects (…) such as [understanding the patient's illness experience]^a^, [incorporating the psychosocial context]^b^ and [ensuring shared decision making]^c^, [considering patients' needs, and including their preferences in care]^a^.

Each unit in square brackets was coded:

a patient as a unique person;

b biopsychosocial perspective;

c patient involvement in care;

### Model development

For the development of the model we specified each dimension's quality by dividing the found dimensions into a) principles (i.e. fundamental propositions, which lay the foundations for patient-centered care), b) enablers (i.e. elements, which foster patient-centered care), and c) activities (i.e. specific patient-centered behavior) of patient-centered care, similar to other work in this field (e.g. [44]–[46]. Furthermore, we mapped the identified dimensions onto different levels of healthcare described in the literature [3], [9], [47]: 1) the micro level, i.e. what takes place inside and around the clinical encounter 2) the meso level, i.e. the level of healthcare institutions, and 3) the macro level, i.e. legislation, policy, payment, regulation and accreditation of healthcare. We discussed the model until we found consensus within the team (IS, JZ, MH, JD).

## Results

### Full texts included

**Figure 1:**
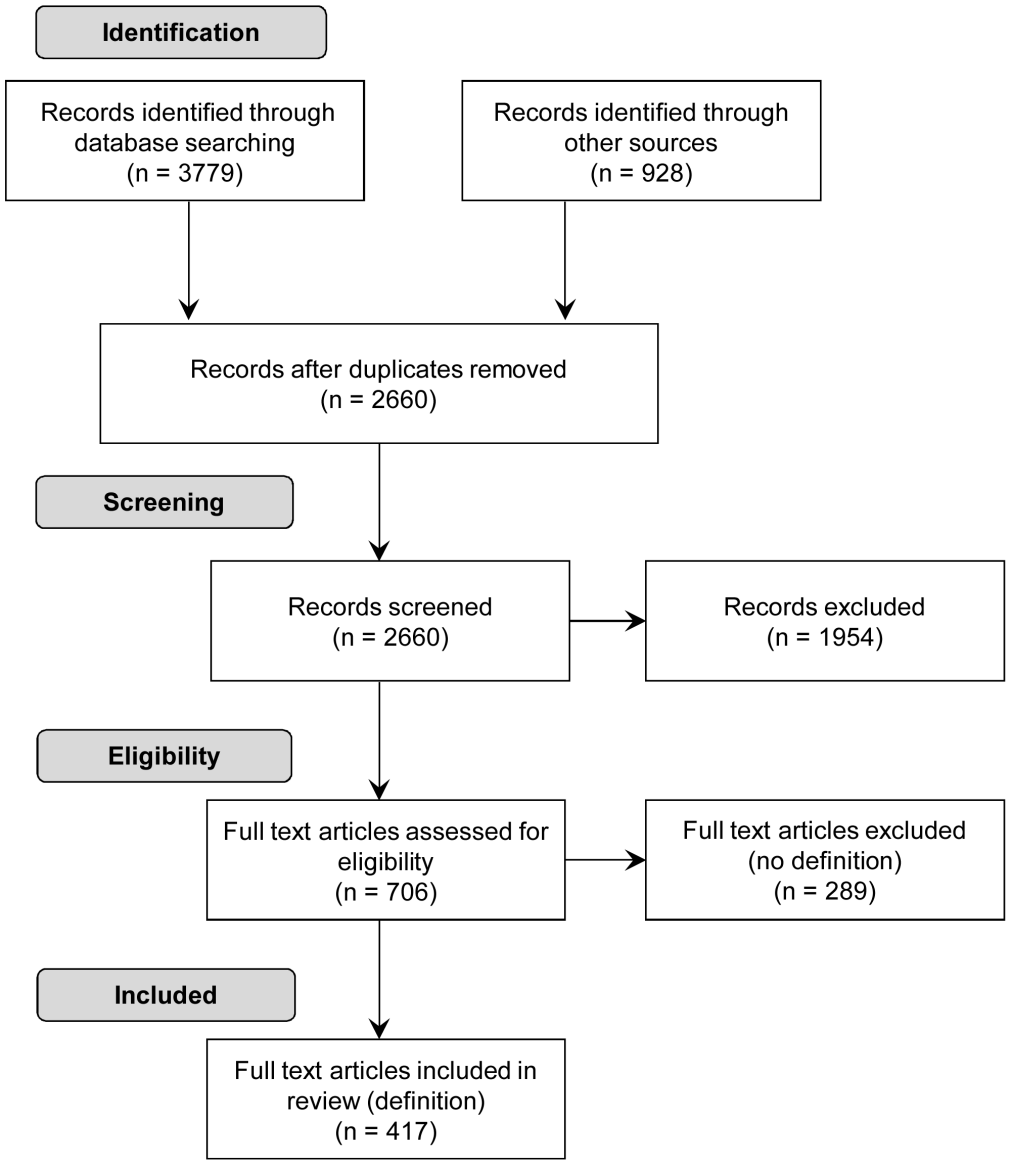
Prisma flow chart of study selection.

A total of 4707 records were identified: 3779 records were generated from the databases search and an additional 928 records were identified through the secondary search. After removing duplicates 2660 records remained. After independent screening by two raters, we retained 706 full texts for further assessment. After examining these full texts, 417 (59%) were retained for concept analysis, see Prisma flow chart in Figure 1. A total of 289 (41%) full texts were excluded at this stage as they did not contain a conceptual definition of patient-centeredness. A list of all 417 full texts included in the analysis is avaible in Appendix S2.

### Characteristics of included full texts

Characteristics of the included full texts are described in Table 2. Approximately 60% of the full texts originated from North America and 33% are from Europe. The included full texts have been published between 1968 and 2012. More than 80% of included full texts have been published after 1999 with the median publication year being 2007.

**Table 2:** Characteristics of included full texts (%).

	Full texts (N = 417)	In %
Countries/regions of origin		
USA	223	53.5
UK & Ireland	62	14.8
Canada	27	6.5
Germany	27	6.5
Netherlands & Belgium	21	5.0
Australia & New Zealand	17	4.1
Scandinavian countries	13	3.1
Other European countries	13	3.1
Asian countries	12	2.9
South Africa	2	0.5
Publication date		
1968–1969	2	0.5
1970–1979	2	0.5
1980–1989	18	4.3
1990–1999	56	13.4
2000–2009	232	55.6
2010 to present	107	25.7

### Dimensions of patient-centeredness

Content analysis of the 417 full texts, which contained definitions of patient-centeredness yielded in 15 dimensions, which are subsequently described. Table 3 gives a brief overview on the dimensions.

**Table 3:** Dimensions of patient-centeredness.

Dimension	Brief description
Principles	
Essential characteristics of the clinician	A set of attitudes towards the patient (e.g. empathy, respect, honesty) and oneself (self-reflectiveness) as well as medical competency
Clinician-patient relationship	A partnership with the patient that is characterized by trust and caring
Patient as a unique person	Recognition of each patient's uniqueness (individual needs, preferences, values, feelings, beliefs, concerns and ideas, and expectations)
Biopsychosocial perspective	Recognition of the patient as a whole person in his or her biological, psychological, and social context
Enablers	
Clinician-patient communication	A set of verbal and nonverbal communication skills
Integration of medical and non-medical care	Recognition and integration of non-medical aspects of care (e.g. patient support services) into health care services
Teamwork and teambuilding	Recognition of the importance of effective teams characterized by a set of qualities (e.g. respect, trust, shared responsibilities, values, and visions) and facilitation of the development of such teams
Access to care	Facilitation of timely access to healthcare that is tailored to the patient (e.g. decentralized services)
Coordination and continuity of care	Facilitation of healthcare that is well coordinated (e.g. regarding follow-up arrangements) and allows continuity (e.g. a well-working transition of care from inpatient to outpatient)
Activities	
Patient information	Provision of tailored information while taking into account the patient's information needs and preferences
Patient involvement in care	Active involvement of and collaboration with the patient regarding decisions related to the patient's health while taking into account the patient's preference for involvement
Involvement of family and friends	Active involvement of and support for the patient's relatives and friends to the degree that the patient prefers
Patient empowerment	Recognition and active support of the patient's ability and responsibility to self-manage his or her disease
	A set of behavior that ensures physical support for the patient (e.g. pain management, assistance with daily living needs)
Emotional support	Recognition of the patient's emotional state and a set of behavior that ensures emotional support for the patient

### Principles

#### Essential characteristics of the clinician

Definitions of patient-centeredness described various qualities that a clinician should have, e.g., being respectful, empathic, tolerant, honest, accountable, compassionate, and committed to the patient. Furthermore, a patient-centered clinician should be self-reflective (e.g. being aware of own emotional responses) and show limited self-disclosure. These qualities should go hand in hand with professional expertise, commitment to evidence based practice, and knowledge of basic psychological skills.

#### Clinician-patient relationship

In the analyzed literature the clinician-patient relationship is described as central for patient-centered care, by building a partnership with the patient through collaboration. The importance of a reciprocal relationship that is characterized by constancy, trust, connection, mutual caring, mutual knowledge, positive rapport building, guidance as well as mutual understanding of roles and responsibilities is highlighted.

#### Patient as a unique person

Another dimension that emerged from analyzing the definitions of patient-centeredness highlights the importance of each patient's uniqueness. This includes eliciting each patient's individual needs, preferences, values, feelings, beliefs, concerns, ideas, and expectations as well as exploring both the patient's disease and illness experience, the impact on functions (e.g. the patient's idea of how the illness affects his or her daily life; effects of the illness on the patient and his or her family), and his or her individual explanatory model. This also entails providing care that is tailored to each specific patient.

#### Biopsychosocial perspective

The dimension biopsychosocial perspective involves understanding the patient's illness within a broader framework by exploring the patient with his or her unique biological, psychological, and social context. This means trying to understand the whole person (e.g. life history, personal and developmental issues), the proximal context (e.g., family, employment, social support, financial situation), as well as the distal context (e.g. cultural background, community, ecosystem) and focusing on the patient's quality of life. In some definitions this entails that the clinicians also feels responsible for non-medical aspects of problems and is involved in the full range of difficulties that the patient brings up.

### Enablers

#### Clinician-patient communication

Many aspects of how to communicate in a patient-centered manner are included in the definitions of patient-centeredness. They include general communication skills, e.g. setting the stage, setting an agenda, prioritizing the patient's problems. A broad range of verbal and non-verbal behavior can be used to engage in patient-centered communication, e.g. using open-ended questions, summarizing important information, asking the patient to repeat, making eye contact, nodding.

#### Integration of medical and non-medical care

Within the definitions of patient-centered care, one element is to integrate medical and non-medical care, i.e. by supporting integrative therapies and complementary medicine, showing sensitivity to non-medical and spiritual dimensions of care, and by offering patient support services (e.g. self-help groups).

#### Teamwork and teambuilding

This dimension recognizes the importance of teamwork and teambuilding for patient-centered care. This has relevance on different levels, e.g. within or between units, departments, healthcare institutions, or between healthcare providers. It can involve building interdisciplinary and multi-skilled teams through training and educational programs. Patient-centered teams are characterized by their ability to communicate, respect and trust among team members, mutually shared values, goals and visions, information sharing, constructive feedback, more equal distribution of responsibility, accountability, and power and awareness of one's own abilities and priorities.

#### Access to care

Furthermore, patient-centeredness includes offering appropriate and preferred access to care, i.e. care that is conveniently located for the patient (e.g. decentralized services, availability of transportation), and that can be accessed in time. It also includes accessibility to specialists or specialty services when a referral is made and provision of clear instructions on when and how to get referrals.

#### Coordination and continuity of care

Last but not least, the literature review showed the importance of coordination and continuity of care to be patient-centered. This includes coordinating front-line patient care with ancillary and support services, and ensuring continuity of care by preparing transitions from inpatient to outpatient or vice-versa and providing follow-up appointment and services after discharge. This dimension also includes making use of known patient data to ensure continuity of care.

### Activities

#### Patient information

This dimension highlights the importance of sharing knowledge and information reciprocally between the clinician and the patient. The clinician should give tailored information (regarding all aspects of care from prevention to treatment, as well as information on how to access medical, psychosocial, physical, and financial support) while eliciting and respecting the patient's information needs and preferences. Some definitions also described the provision of informational resources and tools (e.g. audio records of consultations, multimedia resources, information brochures). Furthermore, the patient should be encouraged to share information (e.g. regarding symptoms and concerns).

#### Patient involvement in care

A prominent dimension often described in the literature on patient-centeredness is the patient's active involvement in care. While older publications use terms like “informed consent” or “sharing power and responsibility”, more recent publications define in more detail the importance of encouraging the patient to participate actively in the consultation and of engaging the patient in the decision making regarding his or her own health (shared decision making). The importance of helping the patient in making informed choices is highlighted in many definitions. This includes respecting the patient's preferences for involvement as well as encouraging the patient's feedback on care (e.g. using patient surveys).

#### Involvement of family and friends

Besides involving the patient in care, some definitions describe the involvement of relatives and friends by providing them with information and involving them in decision making, depending on the patient's preference. It also includes offering support to caregivers and recognizing their needs.

#### Patient empowerment

Another aspect of patient-centered care highlighted in the literature is patient empowerment, by acknowledging the patient's perceived ability to self-manage important aspects of his or her illness, activating and encouraging the patient to take responsibility to solve health related problems and to take actions to improve his or her health and becoming an expert regarding the management of his or her health condition. This also entails supporting the patient's autonomy by offering educational programs, patient activation and health promotion interventions.

#### Physical support

This dimension covers a range of actions that aim at ensuring physical comfort of the patient. This includes pain management, providing assistance with activities and daily living needs (e.g. nutritious food and exercise opportunities during hospital stay), and ensuring safe care (e.g. clean medical facilities).

#### Emotional support

In combination with physical support many definitions of patient-centered care describe the importance of emotional support. This can be achieved by eliciting and responding to emotional issues, paying attention to the patient's anxiety over his or her physical status, treatment and prognosis; anxiety over the impact of the illness on him- or herself and the family; and anxiety over the financial impact of the illness. Other behavior linked to this dimension includes prescribing or recommending medication or psychotherapy to improve the patient's wellbeing if necessary and managing uncertainty by giving information and teaching skills to manage emotions.

### An integrative model of patient-centeredness

The above described 15 dimensions of patient-centeredness found in the literature can be seen as interrelated rather than being independent from one another. For example, the essential characteristics of the clinician influence the clinician-patient-relationship; patient involvement in care is not possible without patient information; emotional support requires good clinician-patient communication; and communication is foundational to build a supportive relationship [48]. This interrelation or overlap of different aspects of patient-centered care has been described in several conceptual descriptions [1], [24] and is reflected in the analyzed literature. The proposed differentiation of the 15 dimensions into a) principles, b) enablers, and c) activities further specifies the interrelation of the identified dimensions. The dimensions patient as a unique person, biopsychosocial perspective, essential characteristics of the clinician and clinician-patient relationship can be seen as underlying principles of patient-centered care. These principles can be implemented by a range of patient-centered activities, i.e. patient information, patient involvement in care, involvement of family and friends, patient empowerment, physical and emotional support. Furthermore, there are certain enablers, which, if present, can be helpful to implement these activities. They are clinician-patient-communication, integration of medical and non-medical care, coordination and continuity of care, access to care and teamwork and team building.

**Figure 2:**
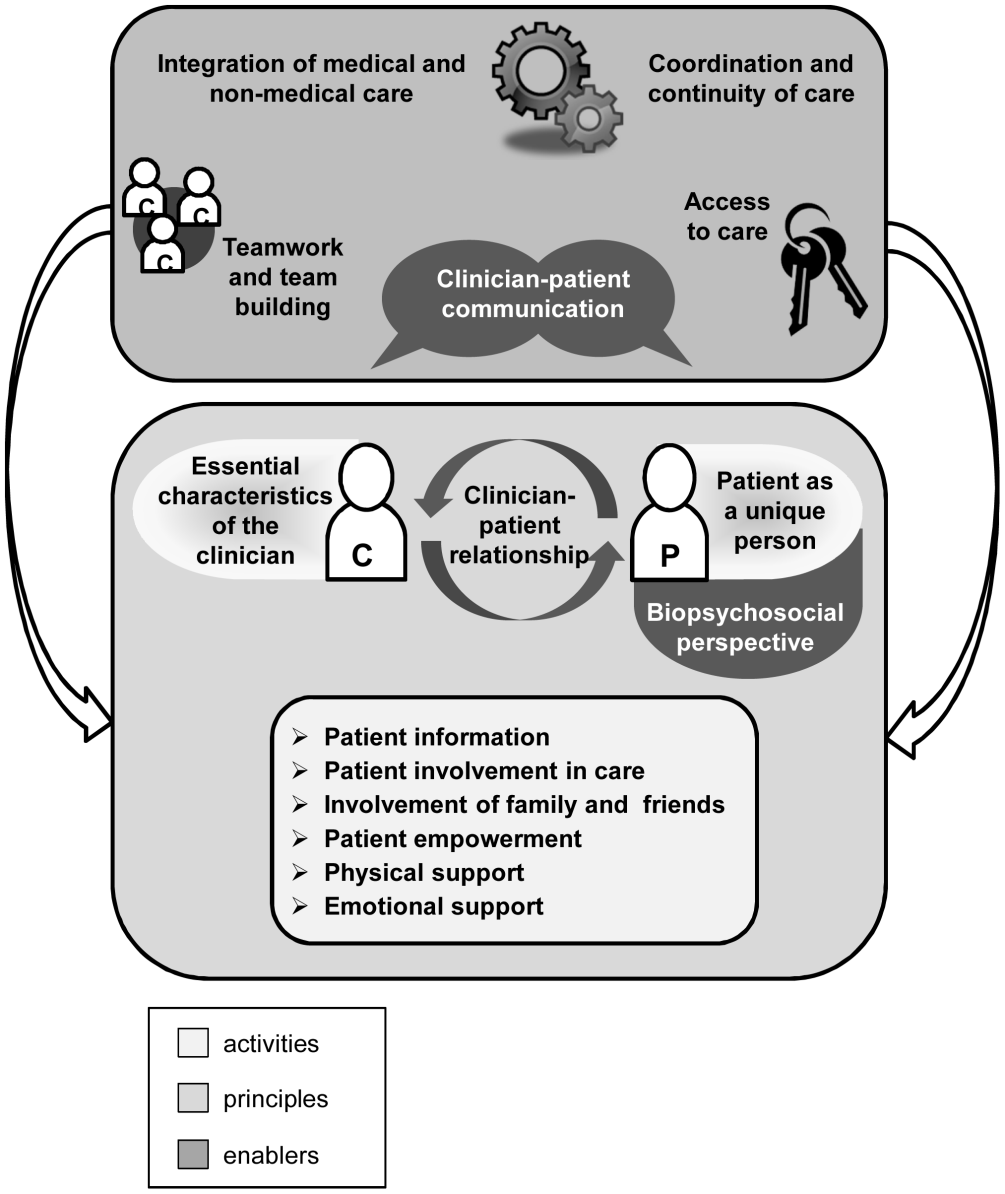
Integrative model of patient-centeredness. The inner circle represents the micro level, the middle circle the meso level and the outer circle the marcro level of care.

Furthermore, the identified dimensions were mapped onto three different levels of healthcare: 1) the micro level, 2) the meso level, and 3) the macro level. While the activities mainly take place on the micro level of care, the enablers are mostly situated on the meso level. None of the dimensions of patient-centeredness identified in the literature focused on the macro level of care. The integrative model of patient-centeredness is displayed in Figure 2.

## Discussion

This study systematically analyzed the different definitions of patient-centeredness found in the literature, identified 15 distinct dimensions of patient-centeredness and proposed an integrative model based on these dimensions. The model highlights that the dimensions are interrelated. They can be divided into principles, enablers and activities. Furthermore, they tackle different levels of care, mainly focusing on the clinical encounter with a patient.

A strength of this study is the use of a systematic review methodology to identify the conceptual definitions in the literature. This allowed to get a broad overview on the different existing definitions and to build an integrative model grounded in a large body of literature of over 400 full texts. In this way, this work adds to previous studies, which were limited to comparing only few definitions [47] or were less comprehensive [23]. A limitation of the study is that the identified dimensions mainly reflect conceptual definitions from North America and Europe. Although some definitions from Australia, New Zealand, Asia, and South Africa were included, further research should investigate whether the identified dimensions of patient-centeredness are applicable to other regions of the world. Another limitation is that this study focused on definitions of patient-centered care and did not take into account synonyms like person-centered care or others. While the term patient-centered care tends to cover a broader range of disease areas [45] and is mainly used in medicine [49], other terms are more predominant in nursing or other specialties [45]. Although these concepts have their origin in different traditions or disciplines, they share their fundamental approach to care [46], [50]. Thus, when comparing the emerging model with definitions of person-centered care, e.g. the definition by the International College of Person-centered Medicine (describing person-centered care as a “medicine of the person, for the person, by the person and with the person [16]), it seems that the difference rather lies in a different nomenclature than in different conceptualizations [51].

The proposed integrative model allows researchers, clinicians, and policy makers to speak the same language. This can have an impact on clinical practice if everyone is on the same page regarding the delivery of patient-centered care. This work provides developers of health policy reports with a comprehensive model of dimensions of patient-centeredness that should be considered if one wants to implement a patient-centered approach to health care in routine practice. As the World Medical Association has recognized, this concept should be more visible and explicitly covered in documents like the Declaration of Lisbon on the Rights of the Patient [52]. The proposed model can also be used in medical and other health care education to design new curricula that have a stronger focus on patient-centeredness [53]. This is in line with the call of the World Federation for Medical Education for a more explicit coverage of the topic in their Global Standards for Medical Education [54]. Furthermore, the proposed integrative model of patient-centeredness provides a foundation for operationalizing the different dimensions of patient-centeredness in future research. It can be used to identify gaps in the measurement of patient-centeredness and eventually to develop new assessment tools to fill these gaps and overcome struggles within the measurement of patient-centeredness [36]. This is a prerequisite for a paradigm shift towards a more patient-centered care, as such a shift needs to be evaluated and monitored. This can only be done by sound measurement tools [16], [53]. At the same time, such a shift needs a change of mind or attitude, as pointed out by the World Medical Association [52]. In order to increase validity of the proposed model, which is based on a comprehensive systematic review, an assessment of its relevance should be conducted including different stakeholders (e.g. clinicians, patients, quality managers), for example in the form of a Delphi study [36]. Furthermore, the mere fact that those 15 dimensions emerge from the literature on patient-centered care does not automatically imply that they lead to positive outcomes for the patients. This should certainly to be subject of further research. However, in order to assess outcomes of certain dimensions of patient-centered care, we need to know which dimensions exist, which was the aim of this study. Finally, the identified dimensions did not relate to the marco level of care, i.e. the analyzed definitions did not contain information on what patient-centeredness means on a health policy and regulation level. However, this seems to be important for the large-scale implementation of patient-centered care into routine practice not only to have enablers on the meso level, but also on the macro level. Certain conditions on this level can function as barriers in delivering patient-centered care, e.g. current reimbursement policies [55] or a progressive move toward specialization [56]. Although prior work has already identified barriers and facilitators for certain patient-centered activities [57], [58], a comprehensive investigation of barriers and facilitators of the identified dimensions of patient-centeredness is necessary in future studies. Patient-centered care can only become reality if barriers on all levels of care have been addressed and ways to overcome them have been found.

## Supporting Information

Appendix S1 **Search strategy in different databases.**
(DOCX)

Appendix S2 **List of included full texts.**
(DOCX)

Checklist S1 **PRISMA Checklist.**
(DOCX)
