# Supplementary material for: An Integrative Model of Patient-Centeredness – A Systematic Review and Concept Analysis
Source: PLoS One. 2014 Sep 17;9(9):e107828. doi: 10.1371/journal.pone.0107828 (PMC4168256; doi:10.1371/journal.pone.0107828)
Supplement: Appendix S1 — Search strategy in different databases. (DOCX) [file pone.0107828.s001.docx]

**Appendix S1: Search strategy in different databases**

| **Medline** |
| --- |
| 1) (patient adj1 centered).m_titl. |
| 2) (patient adj1 centred).m_titl. |
| 3) (patient adj1 centeredness).m_titl. |
| 4) (patient adj1 centredness).m_titl. |
| 5) 1 or 2 or 3 or 4 |
| 6) limit 5 to (English or German) |

| **EMBASE** |
| --- |
| 1) (patient adj1 centered).m_titl. |
| 2) (patient adj1 centeredness).m_titl. |
| 3) (patient adj1 centred).m_titl. |
| 4) (patient adj1 centredness).m_titl. |
| 5) 1 or 2 or 3 or 4 |
| 6) limit 5 to (English or German) |

| **PsycInfo** |
| --- |
| 1) (patient adj1 centered).m_titl. |
| 2) (patient adj1 centeredness).m_titl. |
| 3) (patient adj1 centred).m_titl. |
| 4) (patient adj1 centredness).m_titl. |
| 5) 1 or 2 or 3 or 4 |
| 6) limit 5 to (English or German) |

| **Cochrane Library** |
| --- |
| 1) (patient NEXT centered):ti |
| 2) (patient NEXT centeredness):ti |
| 3) (patient NEXT centred):ti |
| 4) (patient NEXT centredness):ti |
| 5) 1 or 2 or 3 or 4 |

| **Psyndex** |
| --- |
| 1) patientenorientier*.ti. |
| 2) patientenzentrier*.ti |
| 3) 1 or 2 |
| 4) limit 3 to (English or German) |
