# Supplementary material for: An Integrative Model of Patient-Centeredness – A Systematic Review and Concept Analysis
Source: PLoS One. 2014 Sep 17;9(9):e107828. doi: 10.1371/journal.pone.0107828 (PMC4168256; doi:10.1371/journal.pone.0107828)
Supplement: Appendix S2 — List of included full texts. (DOCX) [file pone.0107828.s002.docx]

**Appendix S2: List of included full texts.**

1. Agency for Healthcare Research and Q (2010) Enabling patient-centered care through health information technology (Health IT) (Project record). Agency for Healthcare Research and Quality (AHRQ): Agency for Healthcare Research and Quality (AHRQ).

2. Aita V, McIlvain H, Backer E, McVea K, Crabtree B (2005) Patient-centered care and communication in primary care practice: What is involved? Patient Education & Counseling 58: 296-304.

3. Alamo MM, Moral RR, de Torres LAP (2002) Evaluation of a patient-centred approach in generalized musculoskeletal chronic pain/fibromyalgia patients in primary care. Patient Education and Counseling 48: 23-31.

4. Allen SS, Bland CJ, Dawson SJ (1990) A mini-workshop to train medical students to use a patient- centered approach to smoking cessation. American Journal of Preventive Medicine. pp. 28-33.

5. Anderson EB (2002) Patient-centeredness: A new approach. Nephrology News & Issues 16: 80-82.

6. Anderson GD, Nelson-Becker C, Hannigan EV, Berenson AB, Hankins GDV (2005) A patient-centered health care delivery system by a university obstetrics and gynecology department. Obstetrics & Gynecology 105: 205-210.

7. Anthony MK, Hudson-Barr D (2004) A patient-centered model of care for hospital discharge. Clinical Nursing Research 13: 117-136.

8. Aragon SJ, McGuinn L, Bavin SA, Gesell SB (2010) Does pediatric patient-centeredness affect family trust? Journal for Healthcare Quality 32: 23-31.

9. Arar N, McGrath J, Rosales J, Pugh J (2004) The role of electronic medical records in improving patient-centred care in outpatient encounters. The Journal on Information Technology in Healthcare 2: 187-202.

10. Ashcroft RE (2000) Teaching for patient-centred ethics. Medicine, Health Care & Philosophy 3: 287-295.

11. Audet A-M, Davis K, Schoenbaum SC (2006) Adoption of patient-centered care practices by physicians: Results from a national survey. Archives of Internal Medicine 166: 754-759.

12. Auton GM (1994) The role of total quality management in patient-centered restructuring. Journal of the Society for Health Systems 5: 63-71.

13. Avgar AC, Givan RK, Liu MW (2011) Patient centered but employee delivered: Patient care restructuring, turnover and organizational outcomes in hospitals. Industrial & Labor Relations Review 64: 423-440.

14. Baldwin AS, Cvengros JA, Christensen AJ, Ishani A, Kaboli PJ (2008) Preferences for a patient-centered role orientation: Association with patient-information-seeking behavior and clinical markers of health. Annals of Behavioral Medicine 35: 80-86.

15. Balint E (1969) The possibilities of patient-centered medicine. Journal of the Royal College of General Practitioners 17: 269-276.

16. Bauman AE, Fardy HJ, Harris PG (2003) Getting it right: Why bother with patient-centred care? Medical Journal of Australia 179: 253-256.

17. Beach MC, Rosner M, Cooper LA, Duggan PS, Shatzer J (2007) Can patient-centered attitudes reduce racial and ethnic disparities in care? Academic Medicine 82: 193-198.

18. Belkora JK, Loth MK, Volz S, Rugo HS (2009) Implementing decision and communication aids to facilitate patient-centered care in breast cancer: A case study. Patient Education and Counseling 77: 360-368.

19. Bensing J (2000) Bridging the gap. The separate worlds of evidence-based medicine and patient-centered medicine. Patient Education and Counseling 39: 17-25.

20. Bergvik S, Wynn R, Sorlie T (2008) Nurse training of a patient-centered information procedure for CABG patients. Patient Education and Counseling 70: 227-233.

21. Berlinger N (2008) The nature of chaplaincy and the goals of QI: Patient-centered care as professional responsibility. Hastings Center Report 38: 30-33.

22. Bernstein SB, Zander K (1981) Continuity of care. A patient-centered model. General Hospital Psychiatry 3: 59-63.

23. Berry LL, Seiders K, Wilder SS (2003) Innovations in access to care: A patient-centered approach. Annals of Internal Medicine 139: 568-574.

24. Bertakis KD, Azari R (2011) Patient-centered care is associated with decreased health care utilization. Journal of the American Board of Family Medicine: JABFM 24: 229-239.

25. Bertakis KD, Azari R (2011) Determinants and outcomes of patient-centered care. Patient Education & Counseling 85: 46-52.

26. Bertakis KD, Franks P, Epstein RM (2009) Patient-centered communication in primary care: Physician and patient gender and gender concordance. Journal of Women's Health 18: 539-545.

27. Berwick DM (2009) What 'patient-centered' should mean: Confessions of an extremist. Health Affairs 28: 555-565.

28. Bezold C (2005) The future of patient-centered care: scenarios, visions, and audacious goals. Journal of Alternative & Complementary Medicine 11 Suppl 1: 77-84.

29. Bhugra D, Holsgrove G (2005) Patient-centred psychiatry: Training and assessment: The way forward. Psychiatric Bulletin 29: 49-52.

30. Birks YF, Watt IS (2007) Emotional intelligence and patient-centred care. Journal of the Royal Society of Medicine 100: 368-374.

31. Blanch-Hartigan D, Hall JA, Roter DL, Frankel RM (2010) Gender bias in patients' perceptions of patient-centered behaviors. Patient Education and Counseling. pp. 315-320.

32. Blickem C, Priyadharshini E (2007) Patient narratives: The potential for "patient-centred" interprofessional learning? Journal of Interprofessional Care 21: 619-632.

33. Böck D, Bosch H, Grauhan A, Kohle K, Simons C, et al. (1975) [Continued ecuation for nurses in patient centered nursing/psychosomatic medicine; report of an experience] [German]. Deutsche Krankenpflegezeitschrift 28: 621-626.

34. Bokhour BGP, Mary J., Rao JK, Avetisyan R, Berlowitz DR, Kazis LE (2009) Improving methods for measuring quality of care: A patient-centered approach in chronic disease. Medical Care Research and Review 66: 147-166.

35. Bombeke K, Symons L, Debaene L, De Winter B, Schol S, et al. (2010) Help, I'm losing patient-centredness! Experiences of medical students and their teachers. Medical Education 44: 662-673.

36. Bombeke K, Van Roosbroeck S, De Winter B, Debaene L, Schol S, et al. (2011) Medical students trained in communication skills show a decline in patient-centred attitudes: an observational study comparing two cohorts during clinical clerkships. Patient Education & Counseling 84: 310-318.

37. Bower DJ, Young S, Larson G, Simpson D, Tipnis S, et al. (2009) Characteristics of patient encounters that challenge medical students' provision of patient-centered care. Academic Medicine 84: 74-78.

38. Boyle D, Dwinnell B, Platt F (2005) Invite, Listen, and Summarize: A Patient-Centered Communication Technique. Academic Medicine 80: 29-32.

39. Briggs LA, Kirchhoff KT, Hammes BJ, Song M-K, Colvin ER (2004) Patient-centered advance care planning in special patient populations: A pilot study. Journal of professional nursing : official journal of the American Association of Colleges of Nursing. pp. 47-58.

40. Briggs RW (2011) Clinical decision making for physical therapists in patient-centered end-of-life care. Topics in Geriatric Rehabilitation 27: 10-17.

41. Brown J, Stewart M, McCracken EM, Ian R., Levenstein J (1986) The patient-centred clinical method. 2. definition and application. Family Practice 3: 75-79.

42. Brown JB, Stewart M, McWilliam CL (1999) Using the patient-centered method to achieve excellence in care for women with breast cancer. Patient Education & Counseling 38: 121-129.

43. Brown JB, Stewart M, Ryan BL (2001) Assessing communication between patients and physicians: the measure of patient-centered communication (MPCC) (2e). London, Ontario: University of Western Ontario.

44. Brown JB, Weston WW, Stewart MA (1989) Patient-centred interviewing part II: Finding common ground. Canadian Family Physician 35: 153-157.

45. Brown RF, Bylund CL, Gueguen JA, Diamond C, Eddington J, et al. (2010) Developing patient-centered communication skills training for oncologists: Describing the content and efficacy of training. Communication Education 59: 235-248.

46. Brown SJ (1999) Patient-centered communication. Annual Review of Nursing Research 17: 85-104.

47. Buchholz A, Simon D, Haerter M (2010) Patientenorientierte Gespraechsfuehrung und Fortbildung zur Partizipativen Entscheidungsfindung. Patient-oriented communication and inservice training of participative decision making. Hoefert, Hans-Wolfgang, Haerter, Martin Patientenorientierung im Krankenhaus Goettingen: Hogrefe (2010) S 261-274. pp. 261-274.

48. Buessing A, Glaser J (2003) Mitarbeiter- und Patientenorientierung im Krankenhaus: Implikationen fuer das Qualitaetsmanagement aus Sicht der Arbeitspsychologie. Employee and patient orientation in hospitals: Implications for quality management from the perspective of industrial psychology. Ulich, Eberhard Arbeitspsychologie in Krankenhaus und Arztpraxis Arbeitsbedingungen, Belastungen, Ressourcen Bern: Huber (2003) S 287-304 Series: Schriften zur Arbeitspsychologie, Band 61. pp. 287-304.

49. Buetow SA, Adair V, Coster GDH, M., Gribben B, Mitchell EA (2002) Qualitative insights into practice time management: Does 'patient-centred time' in practice management offer a portal to improved access? British Journal of General Practice 52: 981-987.

50. Cacciatore J (2010) Stillbirth: patient-centered psychosocial care. Clinical Obstetrics & Gynecology 53: 691-699.

51. Calabretta N (2002) Consumer-driven, patient-centered health care in the age of electronic information. Journal of the Medical Library Association 90: 32-37.

52. Calton T, Cheetham A, D'Silva K, Glazebrook C (2009) International schizophrenia research and the concept of patient-centredness: An analysis over two decades. International Journal of Social Psychiatry 55: 157-169.

53. Campinha-Bacote J (2011) Delivering patient-centered care in the midst of a cultural conflict: the role of cultural competence. Online Journal of Issues in Nursing 16: 5.

54. Campion P, Foulkes J, Neighbour R, Tate P (2002) Patient centredness in the MRCGP video examination: analysis of large cohort. Membership of the Royal College of General Practitioners. BMJ 325: 691-692.

55. Carr A, Higginson IJ (2001) Measuring quality of life: Are quality of life measures patient centred? BMJ: British Medical Journal 322: 1357-1360.

56. Carver C, Jessie AT (2011) Patient-centered care in a medical home. Online Journal of Issues in Nursing 16: 4.

57. Cegala DJ, Post DM (2009) The impact of patients' participation on physicians' patient-centered communication. Patient Education and Counseling 77: 202-208.

58. Chapman BP, Duberstein PR, Epstein RM, Fiscella K, Kravitz RL (2008) Patient-centered communication during primary care visits for depressive symptoms: What is the role of physician personality? Medical Care 46: 806-812.

59. Chapman CB (1988) The physician's patient-centered ethical imperative: implications, obligations, and problems. New York State Journal of Medicine 88: 71-73.

60. Charmel PA (2010) Defining and evaluating excellence in patient-centered care. Frontiers of Health Services Management 26: 27-34.

61. Christianson CE, McBride RB, Vari RC, Olson L, Wilson DH (2007) From traditional to patient-centered learning: Curriculum change as an intervention for changing institutional culture and promoting professionalism in undergraduate medical education. Academic Medicine 82: 1079-1088.

62. Clark JG (2007) Patient-centered practice: Aligning professional ethics with patient goals. Seminars in Hearing 28: 163-170.

63. Clayton MF, Dudley WN (2009) Patient-centered communication during oncology follow-up visits for breast cancer survivors: content and temporal structure. Oncology Nursing Forum 36: 68-79.

64. Clayton MF, Latimer S, Dunn TW, Haas L (2011) Assessing patient-centered communication in a family practice setting: How do we measure it, and whose opinion matters? Patient Education & Counseling 84: 294-302.

65. Clifford S, Barber N, Elliott R, Hartley E, Horne R (2006) Patient-centred advice is effective in improving adherence to medicines. Pharmacy World & Science pp. 165-170.

66. Cobb DS (1996) Patient-centered care in an academic health center: Faculty perspective. Journal of Dental Education 60: 955-960.

67. Coffey J, Reagan JT (1984) Assessing patient-centered nursing care. Journal of Long Term Care Administration 12: 25-30.

68. Cohen LA, Bonito AJ, Eicheldinger C, Manski RJ, Macek MD, et al. (2010) Comparison of patient centeredness of visits to emergency departments, physicians, and dentists for dental problems and injuries. Journal of the American College of Dentists 77: 49-58.

69. Cooper K, Smith BH, Hancock E (2008) Patient-centredness in physiotherapy from the perspective of the chronic low back pain patient. Physiotherapy 94: 244–252.

70. Cooper LA, Ford DE, Ghods BK, Roter DL, Primm AB, et al. (2010) A cluster randomized trial of standard quality improvement versus patient-centered interventions to enhance depression care for African Americans in the primary care setting: Study protocol NCT00243425. Implementation Science 5: 1-15.

71. Cousar JB, Peters TH, Jr. (1994) Laboratories in patient-centered units. Clinics in Laboratory Medicine 14: 525-538.

72. Cravens C, Earp JAL (2009) Disclosure and apology: Patient-centered approaches to the public health problem of medical error. North Carolina Medical Journal 70: 140-146.

73. Croom A, Wiebe DJ, Berg CA, Lindsay R, Donaldson D, et al. (2012) Adolescent and parent perceptions of patient-centered communication while managing type 1 diabetes. Journal of Pediatric Psychology 36: 206-215.

74. Cunningham CE, Deal K, Rimas H, Campbell H, Russell A, et al. (2008) Using conjoint analysis to model the preferences of different patient segments for attributes of patient-centered care. The Patient: Patient-Centered Outcomes Research 1: 317-330.

75. Curtis-Tyler K (2011) Levers and barriers to patient-centred care with children: Findings from a synthesis of studies of the experiences of children living with type 1 diabetes or asthma. Child: Care, Health and Development 37: 540-550.

76. Dahm MR (2011) Patient centred care - are international medical graduates 'expert novices'? Australian Family Physician 40: 895-900.

77. Dale J, Sandhu H, Lall R, Glucksman E (2008) The patient, the doctor and the emergency department: A cross-sectional study of patient-centredness in 1990 and 2005. Patient Education and Counseling 72: 320-329.

78. Dancet EAF, Ameye L, Sermeus W, Welkenhuysen M, Nelen WLDM, et al. (2011) The ENDOCARE questionnaire (ECQ): A valid and reliable instrument to measure the patient-centeredness of endometriosis care in Europe. Human Reproduction 26: 2988-2999.

79. Dancet EAF, Van Empel IWH, Rober P, Nelen WLDM, Kremer JAM, et al. (2011) Patient-centred infertility care: A qualitative study to listen to the patient's voice. Human Reproduction 26: 827-833.

80. Dann D, Miller B, Hobbs M, Gentzsch P, Pierson C (1995) Successful interviewing and selection strategies for patient-centered care delivery. Seminars for Nurse Managers 3: 27-35.

81. Das A, Schwartz J, DeRenzo EG (2003) True risk management: physicians' liability risk and the practice of patient-centered medicine. Journal of Law & Health 18: 57-69.

82. Davidson JE, Powers K, Hedayat KM, Tieszen M, Kon AA, et al. (2007) Clinical practice guidelines for support of the family in the patient-centered intensive care unit: American College of Critical Care Medicine Task Force 2004-2005. Critical Care Medicine 35: 605-622.

83. Davidson P, Cockburn J, Daly J, Sanson Fisher R (2004) Patient-centered needs assessment: Rationale for a psychometric measure for assessing needs in heart failure. Journal of Cardiovascular Nursing 19: 164-171.

84. Davis K, Schoenbaum SC, Audet A-M (2005) A 2020 vision of patient-centered primary care. Journal of General Internal Medicine 20: 953-957.

85. Dawood M (2005) Patient centred care: lessons from the medical profession. Emergency Nurse 13: 22-27.

86. de Haes H (2006) Dilemmas in patient centeredness and shared decision making: A case for vulnerability. Patient Education and Counseling 62: 291-298.

87. de Haes H, Koedoot N (2003) Patient centered decision making in palliative cancer treatment: A world of paradoxes. Patient Education and Counseling 50: 43-49.

88. de Lusignan S, Wells S, Russell C (2003) A model for patient-centred nurse consulting in primary care. British Journal of Nursing 12: 85-90.

89. de Oliveira DR, Shoemaker SJ (2006) Achieving patient centeredness in pharmacy practice: Ppenness and the pharmacist's natural attitude. Journal of American Pharmacists Association: JAPhA 46: 56-64.

90. Deen TL, Fortney JC, Pyne JM (2011) Relationship between satisfaction, patient-centered care, adherence and outcomes among patients in a collaborative care trial for depression. Administration and Policy in Mental Health and Mental Health Services Research 38: 345-355.

91. Dick B, Dissen B, Krieg JC, Schreiber W (2001) Qualitaetskriterien der Patientenorientierung in Kliniken und Abteilungen fuer Psychiatrie und Psychotherapie. Die Marburger Modellkriterien nach KTQ(R). Quality criteria of patient orientation in psychiatric hospitals and departments - The Marburg model criteria for KTQ, a hospital top quality management concept. Krankenhauspsychiatrie 12: 145-151.

92. DiGioia A, 3rd, Lorenz H, Greenhouse PK, Bertoty DA, Rocks SD (2010) A patient-centered model to improve metrics without cost increase: Viewing all care through the eyes of patients and families. Journal of Nursing Administration 40: 540-546.

93. Dimick C (2011) First steps to patient-centered care. Journal of Ahima 82: 20-24; quiz 25.

94. Doerge J, Hagenow N (1995) Patient-centered process of work redesign. Arizona hospital reengineers with no outside help. Health Progress 76: 28-32.

95. Dowsett C (2008) Exudate management: A patient-centred approach. Journal of Wound Care 17: 249-252.

96. Dowsett S, Saul J, Butow P, Dunn S, Boyer M, et al. (2000) Communication styles in the cancer consultation: Preferences for a patient-centred approach. Psycho-Oncology 9: 147-156.

97. Drach-Zahavy A (2009) Patient-centred care and nurses' health: The role of nurses' caring orientation. Journal of Advanced Nursing 65: 1463-1474.

98. Dreachslin JL, Hunt PL, Sprainer E (1999) Communication patterns and group composition: Implications for patient-centered care team effectiveness. Journal of Healthcare Management 44: 252-268.

99. Dubé L (2003) What's missing from patient-centered care? Marketing Health Services 23: 30-35.

100. Duffy JR, Lemieux KG (1995) A cardiac service line approach to patient-centered care. Nursing Administration Quarterly 20: 12-23.

101. Duggan PS, Geller G, Cooper LA, Beach MC (2006) The moral nature of patient-centeredness: Is it "just the right thing to do"? Patient Education and Counseling 62: 271-276.

102. Ellis S (1999) The patient-centred care model: holistic/multiprofessional/reflective. British Journal of Nursing 8: 296-301.

103. Ellison D (1968) Patient-centered care in operating room nursing. Nursing Clinics of North America 3: 631-639.

104. Engelhardt K (1971) ["Patient-centred" medicine][German]. Munchener Medizinische Wochenschrift 113: 803-809.

105. Engelhardt K (2003) [Patient-centered medicine and ethics] [German]. Deutsche Medizinische Wochenschrift 128: 1969-1971.

106. Enguidanos SM, Davis C, Katz L (2005) Shifting the paradigm in geriatric care management: Moving from the medical model to patient-centered care. Social Work in Health Care 41: 1-16.

107. Enright SM, Flagstad MS (1994) Patient-centered care: The jury is still out. Topics in Hospital Pharmacy Management 14: 1-6.

108. Epstein RM, Franks P, Fiscella K, Shields CG, Meldrum SC, et al. (2005) Measuring patient-centered communication in Patient-Physician consultations: Theoretical and practical issues. Social Science & Medicine 61: 1516-1528.

109. Epstein RM, Franks P, Shields CG, Meldrum SC, Miller KN, et al. (2005) Patient-centered communication and diagnostic testing. Annals of Family Medicine 3: 415-421.

110. Epstein RM, Street RLJ (2007) Patient-centered communication in cancer care. Promoting healing and reducing suffering. Bethesda, MD: National Cancer Institute.

111. Eriksen HM, Bergdahl J, Bergdahl M (2008) A patient-centred approach to teaching and learning in dental student clinical practice. European Journal of Dental Education 12: 170-175.

112. Evans RG (2003) Patient centred medicine: Reason, emotion, and human spirit? Some philosophical reflections on being with patients. Medical Humanities 29: 8-14.

113. Faller H, Reusch A, Stroebl V, Vogel H (2008) Patientenschulung als Element der Patientenorientierung in der Rehabilitation. Patient education as a constituent of a patient-oriented approach in medical rehabilitation. Die Rehabilitation 47: 77-83.

114. Farin E (2008) Patientenorientierung und ICF-Bezug als Herausforderungen fuer die Ergebnismessung in der Rehabilitation. Patient orientation and reference to the ICF as challenges in rehabilitation outcome assessment. Die Rehabilitation 47: 67-76.

115. Fehrsen GSH, Ronald (1993) In search of excellence: Expanding the patient-centred clinical method: A three-stage assessment. Family Practice 10: 49-54.

116. Fier MA (2007) Patient-centered treatment planning: Part 1. Dentistry Today 26: 56-61.

117. Fisher B, Castle MA, Garrity JM (1998) A cognitive approach to patient-centered abortion care. The new civil war: The psychology, culture, and politics of abortion. Washington, DC: American Psychological Association; US. pp. 301-328.

118. Flach SD, McCoy KD, Vaughn TE, Ward MM, Bootsmiller BJ, et al. (2004) Does patient-centered care improve provision of preventive services? Journal of General Internal Medicine 19: 1019-1026.

119. Fleming NS, Herrin J, Roberts W, Couch C, Ballard DJ (2006) Patient-centeredness and timeliness in a primary care network: Baseline analysis and power assessment for detection of the effects of an electronic health record. Baylor University Medical Center Proceedings 19: 314-319.

120. Flynn K (2006) Systematic reviews for patient-centered care: Update (Structured abstract). Boston: VA Technology Assessment Program (VATAP): VA Technology Assessment Program (VATAP).

121. Fossum B, Arborelius E (2004) Patient-centred communication: Videotaped consultations. Patient Education and Counseling 54: 375-385.

122. Foundation P (2003) Eight dimensions of patient-centered care: Fact sheet.

123. Frampton SB, Guastello S (2010) Patient-centred care: More than the sum of its parts-Planetree's patient-centred hospital designation programme. World Hospitals & Health Services 46: 49-53.

124. Frankel RM, Eddins-Folensbee F, Inui TS (2011) Crossing the patient-centered divide: Transforming health care quality through enhanced faculty development. Academic Medicine 86: 445-452.

125. Freeman TR (1994) The patient-centred case presentation. Family Practice 11: 164-170.

126. Gabrielson A (1997) Patient-centered care in the OR: Is this possible? Canadian Operating Room Nursing Journal 15: 8-10.

127. Gallagher C (2003) 'Parsimonious' versus patient-centered care: Quality issues in childhood immunization. Journal for Healthcare Quality 25: 28-35.

128. Galland L (2006) Patient-centered care: Antecedents, triggers, and mediators. Alternative Therapies in Health & Medicine 12: 62-70.

129. Gambling T, Long AF (2010) The realisation of patient-centred care during a 3-year proactive telephone counselling self-care intervention for diabetes. Patient Education & Counseling 80: 219-226.

130. Gaster B, Edwards K, Trinidad SB, Gallagher TH, Braddock CH, 3rd (2010) Patient-centered discussions about prostate cancer screening: A real-world approach. Annals of Internal Medicine 153: 661-665.

131. Gatterman MI (1995) A patient-centered paradigm: A model for chiropractic education and research. Journal of Alternative & Complementary Medicine 1: 371-386.

132. Geissbuhler A, Spahni S, Assimacopoulos A, Raetzo M-A, Gobet G (2004) Design of a patient-centered, multi-institutional healthcare information network using peer-to-peer communication in a highly distributed architecture. Studies in Health Technology & Informatics 107: 1048-1052.

133. Gerteis M, Edgman-Levitan S, Daley J, Delbanco TL (1993) Through the patient’s eyes. Understanding and Promoting Patient-Centered Care. San Francisco: Jossey-Bass.

134. Gillespie R, Florin D, Gillam S (2004) How is patient-centred care understood by the clinical, managerial and lay stakeholders responsible for promoting this agenda? Health Expectations: An International Journal of Public Participation in Health Care & Health Policy 7: 142-148.

135. Girdley D, Johnsen C, Kwekkeboom K (2009) Facilitating a culture of safety and patient-centered care through use of a clinical assessment tool in undergraduate nursing education. Journal of Nursing Education 48: 702-705.

136. Glasgow RE, Emont S, Miller DC (2006) Assessing delivery of the five 'As' for patient-centered counseling. Health Promotion International 21: 245-255.

137. Godfrey MM, Nelson EC, Wasson JH, Johnson JK, Batalden PB (2007) Planning patient-centered services. Quality by design: A clinical microsystems approach. San Francisco, CA: Jossey-Bass; US. pp. 124-147.

138. Goode CJ, Fink RM, Krugman M, Oman KS, Traditi LK (2010) The Colorado Patient-Centered Interprofessional Evidence-Based Practice Model: A framework for transformation. Worldviews on Evidence-Based Nursing 8: 96-105.

139. Goodrich J (2009) Exploring the wide range of terminology used to describe care that is patient-centred. Nursing Times 105: 14-17.

140. Gorawara-Bhat R, Cook MA (2011) Eye contact in patient-centered communication. Patient Education & Counseling 82: 442-447.

141. Govindarajan P, Larkin GL, Rhodes KV, Piazza G, Byczkowski TL, et al. (2010) Patient-centered integrated networks of emergency care: Consensus-based recommendations and future research priorities. Academic Emergency Medicine 17: 1322-1329.

142. Graugaard PK, Finset A (2000) Trait anxiety and reactions to patient-centered and doctor-centered styles of communication: An experimental study. Psychosomatic Medicine 62: 33-39.

143. Groene O (2011) Patient centredness and quality improvement efforts in hospitals: Rationale, measurement, implementation. International Journal for Quality in Health Care 23: 531-537.

144. Groene O, Lombarts KMJMH, Klazinga NS, Alonso J, Thompson A, et al. (2009) Is patient-centredness in European hospitals related to existing quality improvement strategies? Analysis of a cross-sectional survey (MARQuIS study). Quality and Safety in Health Care 18: i44-50.

145. Grol R, de Maeseneer J, Whitfield M, Mokkink H (1990) Disease-centred versus patient-centred attitudes: Comparison of general practitioners in Belgium, Britain and The Netherlands. Family Practice 7: 100-104.

146. Hahn SR, Friedman DS, Quigley HA, Kotak S, Kim E, et al. (2010) Effect of patient-centered communication training on discussion and detection of nonadherence in glaucoma. Ophthalmology 117: 1339-1347.

147. Haidet P, Fecile ML, West HF, Teal CR (2009) Reconsidering the team concept: Educational implications for patient-centered cancer care. Patient Education & Counseling 77: 450-455.

148. Haidet P, Kelly PA, Bentley S, Blatt B, Chou CL, et al. (2006) Not the same everywhere. Patient-centered learning environments at nine medical schools. Journal of General Internal Medicine 21: 405-409.

149. Haidet PK, P. Adam , Chou C, The Communication CaCSG (2005) Characterizing the patient-centeredness of hidden curricula in medical schools: Development and validation of a new measure. Academic Medicine 80: 44-50.

150. Haisch J, Gundlach G, John M, Stulik A, Reuter T (1989) General practitioners' patient-centered behavior and patients' inferences: Conditions for compliance? Psychotherapie Psychosomatik Medizinische Psychologie 39: 476-479.

151. Hak T, Campion P (1999) Achieving a patient-centred consultation by giving feedback in its early phases. Postgraduate Medical Journal 75: 405-409.

152. Hannich H-J, Wendt M (1983) Patientenzentriertes Verhalten im Umfeld der Operation. Patient-centered treatment of surgery patients. Zeitschrift für personenzentrierte Psychologie und Psychotherapie 2: 311-320.

153. Harkness J (2005) Patient involvement: A vital principle for patient-centred health care. World Hospitals & Health Services 41: 12-16.

154. Hatzichristou D, Tsimtsiou Z (2005) Prevention and management of cardiovascular disease and erectile dysfunction: Toward a common patient-centered, care model. American Journal of Cardiology 96: 80M-84M.

155. Hauer KE, Boscardin C, Gesundheit N, Nevins A, Srinivasan M, et al. (2010) Impact of student ethnicity and patient-centredness on communication skills performance. Medical Education 44: 653-661.

156. Haughton J (2000) A paradigm shift in healthcare. From disease management to patient-centered systems. MD Computing 17: 34-38.

157. Heaven C, Maguire P, Green C (2003) A patient-centred approach to defining and assessing interviewing competency. Epidemiologia e Psichiatria Sociale 12: 86-91.

158. Helitzer DL, Lanoue M, Wilson B, de Hernandez BU, Warner T, et al. (2011) A randomized controlled trial of communication training with primary care providers to improve patient-centeredness and health risk communication. Patient Education and Counseling. pp. 21-29.

159. Helmich P (1984) Patientenorientiertes Arzttum. Utopie oder realisierbare Zukunft? Patient-oriented medical training: Reality or utopia? Praxis der Psychotherapie und Psychosomatik 29: 315-318.

160. Henbest RJ, Fehrsen GS (1992) Patient-centredness: Is it applicable outside the West? Its measurement and effect on outcomes. Family Practice 9: 311-317.

161. Henbest RJ, Stewart M (1990) Patient-centredness in the consultation. 2: Does it really make a difference? Family Practice 7: 28-33.

162. Henbest RJ, Stewart MA (1989) Patient-centredness in the consultation. 1: A method for measurement. Family Practice 6: 249-253.

163. Ho M-J, Yao G, Lee K-L, Hwang T-J, Beach MC (2010) Long-term effectiveness of patient-centered training in cultural competence: What is retained? What is lost? Academic Medicine 85: 660-664.

164. Hobbs JL (2009) A dimensional analysis of patient-centered care. Nursing Research 58: 52-62.

165. Hoefert H-W (2007) Ganzheitlichkeit, Humanitaet und Patientenorientierung - ethische Leitziele oder Marketingbegriffe? Wholeness, humanitarianism, and patient orientation - Ethical goals or marketing concepts? Hoefert, Hans-Wolfgang Fuehrung und Management im Krankenhaus Goettingen: Hogrefe (2007) S 139-162.

166. Hoefert H-W, Haerter M (2010) Einleitung: Patientenorientierung. Introduction: Patient orientation. Hoefert, Hans-Wolfgang, Haerter, Martin Patientenorientierung im Krankenhaus Goettingen: Hogrefe (2010) S 9-30.

167. Holmstrom I, Roing M (2010) The relation between patient-centeredness and patient empowerment: A discussion on concepts. Patient Education & Counseling 79: 167-172.

168. Hudon C, Fortin M, Haggerty JL, Lambert M, Poitras M-E (2011) Measuring patients perceptions of patient-centered care: A systematic review of tools for family medicine. Annals of Family Medicine 9: 155-164.

169. Hunt MR (2009) Patient-centered care and cultural practices: Process and criteria for evaluating adaptations of norms and standards in health care institutions. HEC Forum 21: 327-339.

170. Hutchings HA, Rapport FL, Wright S, Doel MA, Wainwright P (2010) Obtaining consensus regarding patient-centred professionalism in community pharmacy: Nominal group work activity with professionals, stakeholders and members of the public. International Journal of Pharmacy Practice 18: 149-158.

171. Illingworth R (2010) What does 'patient-centred' mean in relation to the consultation? The clinical teacher 7: 116-120.

172. Ishikawa H, Hashimoto H, Roter DL, Yamazaki YT, Tomoko, Yano E (2005) Patient contribution to the medical dialogue and perceived patient-centeredness. An observational study in Japanese geriatric consultations. Journal of General Internal Medicine 20: 906-910.

173. Jacob J (2010) Voice of the patient: The essence of patient-centered care. Critical Care Nursing Clinics of North America 22: 227-232.

174. Jayadevappa R, Chhatre S (2011) Patient centered care - A conceptual model and review of the state of the art. The Open Health Services and Policy Journal 4: 15-25.

175. Jayasinghe UW, Proudfoot J, Holton C, Davies GP, Amoroso C, et al. (2008) Chronically ill Australians' satisfaction with accessibility and patient-centredness. International Journal for Quality in Health Care 20: 105-114.

176. Jorm CM, Dunbar N, Sudano L, Travaglia JF (2009) Should patient safety be more patient centred? Australian Health Review 33: 390-399.

177. Kahn KL, Schneider EC, Malin JL, Adams JL, Epstein AM (2007) Patient centered experiences in breast cancer: Predicting long-term adherence to tamoxifen use. Medical Care 45: 431-439.

178. Kaldy J (2010) Medical home 101: the pharmacist's role in this growing patient-centered care model. Consultant Pharmacist 25: 468-474.

179. Kalkwarf KL (1997) Patient-centered care and today's dental practice. Journal of the American College of Dentists 64: 6-8.

180. Karliner LS, Hwang ES, Nickleach D, Kaplan CP (2011) Language barriers and patient-centered breast cancer care. Patient Education and Counseling 84: 223-228.

181. Kelleher S (2006) Providing patient-centred care in an intensive care unit. Nursing Standard 21: 35-40.

182. Kelly J (2007) Barriers to achieving patient-centered care in Ireland. DCCN - Dimensions of Critical Care Nursing 26: 29-34.

183. Kidd MO, Bond CH, Bell ML (2011) Patients' perspectives of patient-centredness as important in musculoskeletal physiotherapy interactions: a qualitative study. Physiotherapy 97: 154-162.

184. Kinmonth A-L, Spiegal N, Woodcock A (1996) Developing a training programme in patient-centred consulting for evaluation in a randomised controlled trial; diabetes care from diagnosis in British primary care. Patient Education and Counseling. pp. 75-86.

185. Kinmonth A-L, Woodcock A, Griffin S, Spiegal N, Campbell MJ (1998) Randomised controlled trial of patient centred care of diabetes in general practice: impact on current wellbeing and future disease risk. The Diabetes Care From Diagnosis Research Team. BMJ (Clinical research ed). pp. 1202-1208.

186. Kinnersley P, Stott N, Peters TJ, Harvey I (1999) The patient-centredness of consultations and outcome in primary care. British Journal of General Practice 49: 711-716.

187. Kjeldmand D, Holmstrom I, Rosenqvist U (2006) How patient-centred am I? A new method to measure physicians' patient-centredness. Patient Education and Counseling 62: 31-37.

188. Klemperer D (2005) Shared Decision Making und Patientenzentrierung - vom Paternalismus zur Partnerschaft in der Medizin. Teil 1: Modelle der Arzt-Patient-Beziehung. Shared decision-making and patient-centered care - From paternalism to partnership in medicine. Part 1: Models of doctor-patient relations. Balint-Journal 6: 71-79.

189. Koerner M (2010) Patientenorientierte interne Kommunikation im Krankenhaus. Patient-oriented internal communication in hospitals. Hoefert, Hans-Wolfgang, Haerter, Martin Patientenorientierung im Krankenhaus Goettingen: Hogrefe (2010) S 33-50.

190. Kramer M, Schmalenberg C, Maguire P, Brewer BB, Burke R, et al. (2009) Walk the talk: Promoting control of nursing practice and a patient-centered culture. Critical Care Nurse 29: 77-93.

191. Kresevic DM, Counsell SR, Covinsky K, Palmer R, Landefeld CS, et al. (1998) A patient-centered model of acute care for elders. Nursing Clinics of North America 33: 515-527.

192. Krupat E, Bell RA, Kravitz RL, Thom D, Azari R (2001) When physicians and patients think alike: Patient-centered beliefs and their impact on satisfaction and trust. The Journal of Family Practice 50: 1057-1062.

193. Krupat E, Hiam CM, Fleming MZ, Freeman P (1999) Patient-centeredness and its correlates among first year medical students. International Journal of Psychiatry in Medicine 29: 347-356.

194. Kulich KR, Berggren U, Hallberg LRM (2003) A qualitative analysis of patient-centered dentistry in consultations with dental phobic patients. Journal of Health Communication 8: 171-187.

195. Kumagai AK, Murphy EA, Ross PT (2009) Diabetes stories: Use of patient narratives of diabetes to teach patient-centered care. Advances in Health Sciences Education 14: 315-326.

196. Kvale K, Bondevik M (2008) What is important for patient centred care? A qualitative study about the perceptions of patients with cancer. Scandinavian Journal of Caring Sciences 22: 582-589.

197. Lacy NL, Backer EL (2008) Evidence-based and patient-centered care: results from an STFM group project. Family Medicine 40: 417-422.

198. Laine C, Davidoff F (1996) Patient-centered medicine. A professional evolution. JAMA 275: 152-156.

199. Laird-Fick HS, Solomon D, Jodoin C, Dwamena FC, Alexander K, et al. (2010) Training residents and nurses to work as a patient-centered care team on a medical ward. Patient Education & Counseling 84: 90-97.

200. Lambert BL, Street RL, Cegala DJ, Smith DH, Kurtz S, et al. (1997) Provider-patient communication, patient-centered care, and the mangle of practice. Health Communication 9: pp.

201. Lamiani G, Meyer EC, Rider EA, Browning DM, Vegni E, et al. (2008) Assumptions and blind spots in patient-centredness: Action research between american and italian health care professionals. Medical Education 42: 712-720.

202. Langendoen J (2004) The patient-centredness of evidence-based practice. A case example to discuss the clinical application of the bio-psychosocial model. Manual Therapy 9: 228-233.

203. Langer M, Langer N (2009) Hospital implementation of patient-centered communication with aging minority populations. Educational Gerontology 35: 880-889.

204. Langewitz WA, Edlhaimb H-P, Hoefner C, Koschier A, Nuebling M, et al. (2010) Evaluation eines zweijaehrigen Curriculums in Psychosozialer und Psychosomatischer Medizin - Umgang mit Emotionen und patientenzentrierter Gespraechsfuehrung. Evaluation of a two-year curriculum in psychosocial and psychosomatic medicine - Handling emotions and communicating in a patient-centered manner. Psychotherapie, Psychosomatik, Medizinische Psychologie 60: 451-456.

205. Larivaara P, Kiuttu J, Taanila A (2001) The patient-centred interview: The key to biopsychosocial diagnosis and treatment. Scandinavian Journal of Primary Health Care 19: 8-13.

206. Lauver DR, Ward SE, Heidrich SM, Keller ML, Bowers BJ, et al. (2002) Patient-centered interventions. Research in Nursing & Health 25: 246-255.

207. Law SAT, Britten N (1995) Factors that influence the patient centredness of a consultation. British Journal of General Practice 45: 520-524.

208. Laws MB, Epstein L, Lee Y, Rogers W, Beach MC, et al. (2011) The association of visit length and measures of patient-centered communication in HIV care: A mixed methods study. Patient Education & Counseling 85: e183-188.

209. Lecher S, Satzinger W, Trojan A, Koch U (2002) Patientenorientierung durch Patientenbefragungen als ein Qualitaetsmerkmal der Krankenversorgung (PSYNDEXshort). Bundesgesundheitsblatt, Gesundheitsforschung, Gesundheitsschutz 45: 3-12.

210. Lee Y-YL, Julia L. (2010) Do patient autonomy preferences matter? Linking patient-centered care to patient-physician relationships and health outcomes. Social Science & Medicine 71: 1811-1818.

211. Lehmann H (1986) Patientenorientierung in der Herzinfarktrehabilitation. Patient orientation in myocardial infarction rehabilitation. Silomon, Hero, Brennecke, Ralph, von Ferber, Christian, Laaser, Ulrich Sozialmedizin, Sozialrecht, Gesundheitsoekonomie Berlin: Springer (1986) Seiten 152-161.

212. Lein C, Wills CE (2007) Using patient-centered interviewing skills to manage complex patient encounters in primary care. Journal of the American Academy of Nurse Practitioners 19: 215-220.

213. Leung FW (2008) Promoting informed choice of unsedated colonoscopy: patient-centered care for a subgroup of US Veterans. Digestive Diseases & Sciences 53: 2955-2959.

214. Leung GM, Fielding R, Chan M-F, Lee A, Cheng Y-HY, Clara, et al. (2002) The development and evaluation of an integrated community-based, patient-centred learning activity at the University of Hong Kong. Medical Education 36: 992-995.

215. Levenstein JH, McCracken EC, McWhinney IR, Stewart MA, Brown JB (1986) The patient-centred clinical method. 1. A model for the doctor-patient interaction in family medicine. Family Practice 3: 24-30.

216. Levinson W, Lesser CS, Epstein RM (2010) Developing physician communication skills for patient-centered care. Health Affairs 29: 1310-1318.

217. Lewin S, Skea Z, Entwistle Vikki A, Zwarenstein M, Dick J (2001) Interventions for providers to promote a patient-centred approach in clinical consultations. Cochrane Database of Systematic Reviews. Chichester, UK: John Wiley & Sons, Ltd.

218. Little P, Everitt H, Williamson I, Warner G, Moore M, et al. (2001) Observational study of effect of patient centredness and positive approach on outcomes of general practice consultations. BMJ: British Medical Journal 323: 908-911.

219. Lowes R (1998) Patient-centered care for better patient adherence. Family Practice Management 5: 46-57.

220. Lown BAM, Colleen F. (2010) The Schwartz Center Rounds: Evaluation of an interdisciplinary approach to enhancing patient-centered communication, teamwork, and provider support. Academic Medicine 85: 1073-1081.

221. Lutz BJ, Bowers BJ (2000) Patient-centered care: Understanding its interpretation and implementation in health care. Scholarly Inquiry for Nursing Practice 14: 165-187.

222. Luxford K, Safran DG, Delbanco T (2011) Promoting patient-centered care: A qualitative study of facilitators and barriers in healthcare organizations with a reputation for improving the patient experience. International Journal for Quality in Health Care 23: 510-515.

223. Madhan BR, Ajay S. , Gayathri H (2011) Attitudes of postgraduate orthodontic students in India towards patient-centered care. Journal of Dental Education 75: 107-114.

224. Magnil M, Gunnarsson R, Bjoorkelund C (2011) Using patient-centred consultation when screening for depression in elderly patients: A comparative pilot study. Scandinavian Journal of Primary Health Care 29: 51-56.

225. Maizes V, Rakel D, Niemiec C (2009) Integrative medicine and patient-centered care. Explore: The Journal of Science & Healing 5: 277-289.

226. Mallinger JB, Griggs JJ, Shields CG (2005) Patient-centered care and breast cancer survivors' satisfaction with information. Patient Education and Counseling 57: 342-349.

227. Mansur JM, Chamerlik SJ, Bohenek W, Leonard TM, Dean RP (1994) Involvement of a pharmacy department in a hospital's transition to patient-centered care. Topics in Hospital Pharmacy Management 14: 36-45.

228. Martin D (2003) Martin's Map: A conceptual framework for teaching and learning the medical interview using a patient-centred approach. Medical Education 37: 1145-1153.

229. Martin DP, Diehr P, Conrad DA, Davis JH, Leickly R, et al. (1998) Randomized trial of a patient-centered hospital unit. Patient Education and Counseling 34: 125-133.

230. May C, Mead N, Bower P (1999) Patient-centeredness: A history. In: Dowrick C, Frith L, editors. General practice and ethics: Uncertainty and responsibility. London: Routledge. pp. 76-90.

231. McClimans LM, Dunn M, Slowther A-M (2011) Health policy, patient-centred care and clinical ethics. Journal of Evaluation in Clinical Practice 17: 913-919.

232. McCormack LA, Treiman K, Rupert D, Williams-Piehota P, Nadler E, et al. (2011) Measuring patient-centered communication in cancer care: A literature review and the development of a systematic approach. Social Science & Medicine 72: 1085-1095.

233. McCracken EC, Stewart MA, Brown JB, McWhinney IR (1983) Patient-centred care: The family practice model. Canadian Family Physician 29: 2313-2316.

234. McKeon LM, Norris T, Cardell B, Britt T (2009) Developing patient-centered care competencies among prelicensure nursing students using simulation. Journal of Nursing Education 48: 711-715.

235. McKinley S, Elliott RM (2008) Implications for Australian practice of North American guidelines for the support of the family in patient-centred intensive care. Collegian: Journal of the Royal College of Nursing, Australia 15: 11-17.

236. McKinnon J (2007) Patient-Centred Planning and Concordance. Towards prescribing practice. New York, NY: John Wiley & Sons Ltd; US. pp. 35-57.

237. McPherson T, Fontane P (2010) Patient-centered care in the community-based compounding practice setting. Journal of the American Pharmacists Association: JAPhA 50: 37-44.

238. McWhinney IR (1985) Patient-centered and doctor-centered models of clinical decision making. In: Sheldon M, Brook J, Rector A, editors. Decision making in general practice. London: Stockton. pp. 31-46.

239. Mead N, Bower P (2000) Measuring patient-centredness: A comparison of three observation-based instruments. Patient Education and Counseling 39: 71-80.

240. Mead N, Bower P (2000) Patient-centredness: A conceptual framework and review of the empirical literature. Social Science & Medicine 51: 1087-1110.

241. Mead N, Bower P (2002) Patient-centred consultations and outcomes in primary care: A review of the literature. Patient Education and Counseling 48: 51-61.

242. Mead N, Bower P, Hann M (2002) The impact of general practitioners' patient-centredness on patients' post-consultation satisfaction and enablement. Social Science & Medicine 55: 283-299.

243. Medicine Io (2001) Crossing on quality chasm: A new health system for the 21st century. Washington D.C.: National Academy Press.

244. Meffert H-J (1988) Patientenzentrierte psychologische Arbeit in der Herzchirurgie. Patient-centered consultation-liaison psychology with heart surgery patients. Gesellschaft fuer wissenschaftliche Gespraechspsychotherapie Orientierung an der Person Band 1: Diesseits von Psychotherapie 7 Symposion der GwG vom 10-12 Oktober 1986 in Koeln Koeln: Gesellschaft fuer wissenschaftliche Gespraechspsychotherapie (1988) Seiten 91-94.

245. Meterko M, Wright S, Lin H, Lowy E, Cleary PD (2010) Mortality among patients with acute myocardial infarction: the influences of patient-centered care and evidence-based medicine. Health Services Research 45: 1188-1204.

246. Michie S, Miles J, Weinman J (2003) Patient-centredness in chronic illness: What is it and does it matter? Patient Education and Counseling 51: 197-206.

247. Miller E (1999) Reengineering the role of a nurse manager in a patient-centered care organization. Journal of Nursing Care Quality 13: 47-56.

248. Miller NA (1997) Patient centered long-term care. Health Care Financing Review 19: 1-10.

249. Montgomery K, Little M (2011) Enriching patient-centered care in serious illness: A focus on patients' experiences of agency. Milbank Quarterly 89: 381-398.

250. Moore M (2008) What does patient-centred communication mean in Nepal? Medical Education 42: 18-26.

251. Moore M (2009) What do Nepalese medical students and doctors think about patient-centred communication? Patient Education and Counseling 76: 38-43.

252. Moral RR, Alamo MM, Jurado MA, de Torres LP (2001) Effectiveness of a learner-centred training programme for primary care physicians in using a patient-centred consultation style. Family Practice 18: 60-63.

253. Moran J, Bekker H, Latchford G (2008) Everyday use of patient-centred, motivational techniques in routine consultations between doctors and patients with diabetes. Patient Education and Counseling 73: 224-231.

254. Morgan M, Dodds W, Wolfe C, Raju S (2004) Women's views and experiences of outpatient hysteroscopy: Implications for a patient-centered service. Nursing & Health Sciences 6: 315-320.

255. Morris J, Leonard R (2007) Physiotherapy students' experiences of palliative care placements-Promoting interprofessional learning and patient-centred approaches. Journal of Interprofessional Care 21: 569-571.

256. Moss AH (2001) Patient-centered medicine: Achieving outcomes in end-of-life care that will meet with high patient and family satisfaction. West Virginia Medical Journal 97: 306-307.

257. Murdoch JC (2003) Chronic fatigue syndrome. The patient centred clinical method-a guide for the perplexed. Australian Family Physician 32: 883-887.

258. Murphy J (2011) Patient as center of the health care universe: A closer look at patient-centered care. Nursing Economics 29: 35-37.

259. Murphy SAR, Freed JS (2008) Healthcare's ascension to patient-centered genomic care. Personalized Medicine 5: 505-509.

260. Nailon RE (2007) The assessment and documentation of language and communication needs in healthcare systems: Current practices and future directions for coordinating safe, patient-centered care. Nursing Outlook 55: 311-317.

261. Napier B (1998) Diversity and aging. Cultural understanding as a powerful force in patient-centered healing. Home Care Provider 3: 38-40.

262. Nazario RJ (2009) Medical humanities as tools for the teaching of patient-centered care. Journal of Hospital Medicine (Online) 4: 512-514.

263. Nestel D (2001) Evaluation of a communication skills course: Cultural relevance of the patient-centred interview in a Hong Kong Chinese setting. Medical Teacher 23: 212-214.

264. Nestel D, Betson C (1999) An evaluation of a communication skills workshop for dentists: Cultural and clinical relevance of the patient-centred interview. British Dental Journal 187: 385-388.

265. Ngo-Metzger Q, August KJ, Srinivasan M, Liao S, Meyskens FL, Jr. (2008) End-of-Life care: Guidelines for patient-centered communication. American Family Physician 77: 167-174.

266. Nicolaidis C (2011) Police officer, deal-maker, or health care provider? Moving to a patient-centered framework for chronic opioid management. Pain Medicine 12: 890-897.

267. Nobili MP, Piergrossi S, Brusati V, Moja EA (2007) The effect of patient-centered contraceptive counseling in women who undergo a voluntary termination of pregnancy. Patient Education and Counseling 65: 361-368.

268. Nordehn G, Meredith A, Bye L (2006) A preliminary investigation of barriers to achieving patient-centered communication with patients who have stroke-related communication disorders. Topics in Stroke Rehabilitation 13: 68-77.

269. Ockene JK, Ockene IS, Quirk ME, Hebert JR, Saperia GM, et al. (1995) Physician training for patient-centered nutrition counseling in a lipid intervention trial. Preventive Medicine 24: 563-570.

270. Ockene JK, Wheeler EV, Adams A, Hurley TG, Hebert J (1997) Provider training for patient-centered alcohol counseling in a primary care setting. Archives of internal medicine. pp. 2334-2341.

271. O'Donovan A (2007) Patient-centred care in acute psychiatric admission units: Reality or rhetoric? Journal of Psychiatric and Mental Health Nursing 14: 542-548.

272. O'Flynn N, Britten N (2006) Does the achievement of medical identity limit the ability of primary care practitioners to be patient-centred? A qualitative study. Patient Education and Counseling 60: 49-56.

273. Ogden J, Ambrose L, Khadra AM, Sushma, Symons LV, Alex

Williams, Michelle (2002) A questionnaire study of GPs' and patients' beliefs about the different components of patient centredness. Patient Education and Counseling 47: 223-227.

274. O'Keefe M, Roberton DS, Michael, Baghurst P (2003) Medical student interviewing: A randomized trial of patient-centredness and clinical competence. Family Practice. pp. 213-219.

275. Olsson L-E, Hansson E, Ekman I, Karlsson J (2009) A cost-effectiveness study of a patient-centred integrated care pathway. Journal of Advanced Nursing 65: 1626-1635.

276. Ouwens M, Hermens R, Hulscher MV-O, Saskia

Tjan-Heijnen, Vivianne, Termeer R, Marres H, et al. (2010) Development of indicators for patient-centred cancer care. SUPPORTIVE CARE IN CANCER 18: 121-130.

277. Ozmon J (2007) Consumerism: Forcing medical practices toward patient-centered care. Journal of Medical Practice Management 23: 44-46.

278. Pardon K, Deschepper R, Vander Stichele R, Bernheim J, Mortier F, et al. (2009) Preferences of advanced lung cancer patients for patient-centred information and decision-making: A prospective multicentre study in 13 hospitals in Belgium. Patient Education and Counseling 77: 421-429.

279. Passalacqua SA, Segrin C (2012) The Effect of Resident Physician Stress, Burnout, and Empathy on Patient-Centered Communication During the Long-Call Shift. Health Communication 27: 449-456.

280. Pearce C, Trumble S (2006) Computers can't listen--algorithmic logic meets patient centredness. Australian Family Physician 35: 439-442.

281. Pelzang R (2010) Time to learn: Understanding patient-centred care. British Journal of Nursing 19: 912-917.

282. Pelzang R, Wood B, Black S (2010) Nurses' understanding of patient-centred care in Bhutan. British Journal of Nursing 19: 186-193.

283. Perlin JB, Kolodner RM, Roswell RH (2005) The Veterans Health Administration: Quality, value, accountability, and information as transforming strategies for patient-centered care. Healthcarepapers 5: 10-24.

284. Perocchia RS, Hodorowski JK, Williams LA, Kornfeld J, Davis NL, et al. (2010) Patient-centered communication in cancer care: The role of the NCI's Cancer Information Service. Journal of Cancer Education 26: 36-43.

285. Pfeiffer WM (1988) Thesen zur Anregung einer Diskussion ueber Probleme patientenzentrierter Medizin. Some theses as an introduction to a discussion on the problems of patient-centered medicine. Gesellschaft fuer wissenschaftliche Gespraechspsychotherapie Orientierung an der Person Band 1: Diesseits von Psychotherapie 7 Symposion der GwG vom 10-12 Oktober 1986 in Koeln Koeln: Gesellschaft fuer wissenschaftliche Gespraechspsychotherapie (1988) Seiten 85-86.

286. Pham JC, Trueger NS, Hilton JK, Rahul K., Smith JP, Bernstein SL (2011) Interventions to improve patient-centered care during times of emergency department crowding. Academic Emergency Medicine 18: 1289-1294.

287. Piccolo LD, Mazzi MA, Scardoni S, Gobbi M, Zimmermann C (2008) A theory-based proposal to evaluate patient-centred communication in medical consultations: The Verona Patient-centred Communication Evaluation scale (VR-COPE). Health Education 108: 355-372.

288. Platt FW, Gaspar DL, Coulehan JL, Fox L, Adler AJ, et al. (2001) "Tell me about yourself": The patient-centered interview. Annals of Internal Medicine 134: 1079-1085.

289. Pollock K, Grime J (2003) The cost and cost-effectiveness of PPIs--GP perspectives and responses to a prescribing dilemma and their implications for the development of patient-centred healthcare. European Journal of General Practice 9: 126-133.

290. Ponte PR, Conlin G, Conway JB, Grant S, Medeiros C, et al. (2003) Making patient-centered care come alive: Achieving full integration of the patient's perspective. Journal of Nursing Administration 33: 82-90.

291. Poochikian-Sarkissian S, Wennberg RA, Sidani S (2008) Examining the relationship between patient-centred care and outcomes on a neuroscience unit: A pilot project. Canadian Journal of Neuroscience Nursing 30: 14-19.

292. Prueksaritanond S, Tubtimtes S, Asavanich K, Tiewtranon V (2004) Type 2 diabetic patient-centered care. Journal of the Medical Association of Thailand 87: 345-352.

293. Radwin LE (2003) Cancer patients' demographic characteristics and ratings of patient-centered nursing care. Journal of Nursing Scholarship 35: 365-370.

294. Radwin LE, Cabral HJ, Wilkes G (2009) Relationships between patient-centered cancer nursing interventions and desired health outcomes in the context of the health care system. Research in Nursing & Health 32: 4-17.

295. Rainone F, McHugh M (2007) Patient-centered palliative care in the home. Choices in palliative care: Issues in health care delivery. New York, NY: Springer Science + Business Media; US. pp. 31-44.

296. Ralston JD, Martin DP, Anderson ML, Fishman PA, Conrad DA, et al. (2009) Group health cooperative's transformation toward patient-centered access. Medical Care Research & Review 66: 703-724.

297. Reddy M, Kohr R, Queen D, Keast D, Sibbald RG (2003) Practical treatment of wound pain and trauma: A patient-centered approach. An overview. Ostomy Wound Management 49: 2-15.

298. Redfern J, McKevitt C, Wolfe CD (2006) Risk management after stroke: The limits of a patient-centred approach. Health, Risk & Society 8: 123–141.

299. Redman RW (2004) Patient-Centered Care: An Unattainable Ideal? Research and Theory for Nursing Practice: An International Journal 18: 11-14.

300. Redman RW (2008) Whither patient-centered care? Research and Theory for Nursing Practice: An International Journal 22: 5-6.

301. Redman RW, Jones KR (1998) Effects of implementing patient-centered care models on nurse and non-nurse managers. Journal of Nursing Administration 28: 46-53.

302. Redman RW, Lynn MR (2004) Advancing patient-centred care through knowledge development. Canadian Journal of Nursing Research 36: 116-129.

303. Regan-Smith M, Hirschmann K, lobst W (2007) Direct observation of faculty with feedback: An effective means of improving patient-centered and learner-centered teaching skills. Teaching and Learning in Medicine 19: 278-286.

304. Reti SR, Feldman HJ, Ross SE, Safran C (2010) Improving personal health records for patient-centered care. Journal of the American Medical Informatics Association 17: 192-195.

305. Reynolds A (2009) Patient-centered care. Radiologic Technology 81: 133-147.

306. Rhodes P, Langdon M, Rowley E, Wright J, Small N (2006) What does the use of a computerized checklist mean for patient-centered care? The example of a routine diabetes review. Qualitative Health Research. Qualitative Health Research 16: 353-376.

307. Richards PS, Inglehart MR (2006) An interdisciplinary approach to case-based teaching: Does it create patient-centered and culturally sensitive providers? Journal of Dental Education. pp. 284-291.

308. Rimondini M, Del Piccolo L, Goss C, Mazzi MA, Paccaloni M, et al. (2010) The evaluation of training in patient-centred interviewing skills for psychiatric residents. Psychological Medicine 40: 467-476.

309. Ripke T (1987) Patientenorientierte koerperliche Untersuchung. Patient-centered medical examination. Zeitschrift für personenzentrierte Psychologie und Psychotherapie 6: 185-192.

310. Ripke T (1994) "Rezepte" fuer patientenorientiertes Handeln im Verlauf des Allgemeinpraxisgespraechs. Suggestions for general practitioners' patient-oriented behavior in conversations with the patient. Geyer, Michael, Hirsch, Reinhard Psychotherapie in der Psychosomatischen Grundversorgung Leipzig: Barth (1994) Seiten 46-51 Series: Psychotherapeutische Medizin, Band 1.

311. Rittenhouse DR, Thom DH, Schmittdiel JA (2010) Developing a policy-relevant research agenda for the patient-centered medical home: A focus on outcomes. Journal of General Internal Medicine 25: 593-600.

312. Rivadeneyra R, Elderkin-Thompson V, Silver RC, Waitzkin H (2000) Patient centeredness in medical encounters requiring an interpreter. American Journal of Medicine 108: 470-474.

313. Robinson JH, Callister LC, Berry JA, Dearing KA (2008) Patient-centered care and adherence: Definitions and applications to improve outcomes. Journal of the American Academy of Nurse Practitioners 20: 600-607.

314. Robinson L, Bamford C, Briel R, Spencer J, Whitty P (2010) Improving patient-centered care for people dementia in medical encounters: An educational intervention for old age psychiatrists. International Psychogeriatrics 22: 129-138.

315. Robinson NC (1991) A patient-centered framework for restructuring care. Journal of Nursing Administration 21: 29-34.

316. Rocco N, Scher K, Basberg B, Yalamanchi S, Baker-Genaw K (2011) Patient-centered plan-of-care tool for improving clinical outcomes. Quality Management in Health Care 20: 89-97.

317. Rogers A, Kennedy A, Nelson E, Robinson A (2005) Uncovering the limits of patient-centeredness: Implementing a self-management trial for chronic illness. Qualitative Health Research. pp. 224-239.

318. Rosal MC, Ebbeling CB, Lofgren I, Ockene JK, Ockene IS, et al. (2001) Facilitating dietary change: the patient-centered counseling model. Journal of the American Dietetic Association 101: 332-341.

319. Ross EF, Haidet P (2011) Attitudes of physical therapy students toward patient-centered care, before and after a course in psychosocial aspects of care. Patient Education & Counseling 85: 529-532.

320. Roter DL, Hall JA (2004) Physician gender and patient-centered communication: A critical review of empirical research. Annual Review of Public Health 25: 497-519.

321. Roter DL, Larson S, Sands DZ, Ford DE, Houston T (2008) Can e-mail messages between patients and physicians be patient-centered? Health Communication 23: 80-86.

322. Rouf E, Chumley H, Dobbie A (2009) Patient-centered interviewing and student performance in a comprehensive clinical skills examination: Is there an association? Patient Education and Counseling 75: 11-15.

323. Roumie CL, Greevy R, Wallston KA, Elasy TA, Kaltenbach L, et al. (2011) Patient centered primary care is associated with patient hypertension medication adherence. Journal of Behavioral Medicine 34: 244-253.

324. Saenger S (2010) Patientenorientiertes Qualitaetsmanagement. Patient-oriented quality management. Hoefert, Hans-Wolfgang, Haerter, Martin Patientenorientierung im Krankenhaus Goettingen: Hogrefe (2010) S 51-77.

325. Saha S, Beach MC, Cooper LA (2008) Patient centeredness, cultural competence and healthcare quality. Journal of the National Medical Association 100: 1275-1285.

326. Saha SB, Mary Catherine (2011) The impact of patient-centered communication on patients' decision making and evaluations of physicians: A randomized study using video vignettes. Patient Education & Counseling 84: 386-392.

327. Schaeffer D (1993) Patientenorientierung und Gesundheitsfoerderung im Akutkrankenhaus. Erfordernisse der Organisations- und Strukturentwicklung. Patient orientation and health promotion in acute hospitals: Results of organizational and structural development. Pelikan, Juergen M, Demmer, Hildegard, Hurrelmann, Klaus Gesundheitsfoerderung durch Organisationsentwicklung Konzepte, Strategien und Projekte fuer Betriebe, Krankenhaeuser und Schulen Weinheim: Juventa (1993) Seiten 267-284.

328. Schaeffer D (1995) Patientenorientierte Krankenversorgung: AIDS als Herausforderung. Patient-oriented health care: AIDS as a challenge. Zeitschrift fuer Gesundheitswissenschaften 3: 332-348.

329. Schall M, Sevin C, Wasson JH (2009) Making high-quality, patient-centered care a reality. Journal of Ambulatory Care Management 32: 3-7.

330. Schoenle PW (2003) Anforderungen an eine patientenorientierte Rehabilitation. Demands on a new, patient-oriented rehabilitation. Die Rehabilitation 42: 261-268.

331. Scholl JC (2007) The use of humor to promote patient-centered care. Journal of Applied Communication Research 35: 156-176.

332. Sepucha KR, Fowler FJ, Jr., Mulley AG, Jr. (2004) Policy support for patient-centered care: The need for measurable improvements in decision quality. Health Affairs Suppl Web Exclusives: VAR54-62.

333. Sevin C, Moore G, Shepherd J, Jacobs T, Hupke C (2009) Transforming care teams to provide the best possible patient-centered, collaborative care. Journal of Ambulatory Care Management 32: 24-31.

334. Sherer JL (1994) Putting patients first. Hospitals work to define patient-centered care. Trustee 47: 14-16.

335. Shields CG, Epstein RM, Fiscella K, Franks P, McCann R, et al. (2005) Influence of accompanied encounters on patient-centeredness with older patients. The Journal of the American Board of Family Practice / American Board of Family Practice. pp. 344-354.

336. Shields CGC, Casey J., Poulsen SS, Doyle JM, Fiscella K, Epstein RM, et al. (2009) Patient-centered communication and prognosis discussions with cancer patients. Patient Education and Counseling 77: 437-442.

337. Sidani S (2008) Effects of patient-centered care on patient outcomes: An evaluation. Research and Theory for Nursing Practice: An International Journal 22: 24-37.

338. Sidani S, Epstein D, Miranda J (2006) Eliciting patient treatment preferences: A strategy to integrate evidence-based and patient-centered care. Worldviews on Evidence-Based Nursing 3: 116-123.

339. Sine DM, Sharpe VA (2011) Ethics, risk, and patient-centered care: How collaboration between clinical ethicists and risk management leads to respectful patient care. Journal of Healthcare Risk Management 31: 32-37.

340. Skinder-Meredith A, Bye L, Bulthuis K, Schueller A (2007) Patient-centered communication survey of nursing homes and rehabilitation centers. Care Management Journals 8: 8-15.

341. Small DC, Small RM (2011) Patients first! Engaging the hearts and minds of nurses with a patient-centered practice model. Online Journal of Issues in Nursing 16: 15.

342. Smith DH, Dixon AS, Lam CL, Lam T-P (1999) Patient-centered communication in Hong Kong. Health Communication 11: 285-297.

343. Smith F, Orrell M (2007) Does the patient-centred approach help identify the needs of older people attending primary care? Age & Ageing 36: 628-631.

344. Smith RC, Dorsey AM, Lyles JS, Frankel RM (1999) Teaching self-awareness enhances learning about patient-centered interviewing. Academic Medicine 74: 1242-1248.

345. Smith RC, Dwamena FC, Grover M, Coffey J, Frankel RM (2011) Behaviorally defined patient-centered communication--A narrative review of the literature. Journal of General Internal Medicine 26: 185-191.

346. Smith RC, Marshall-Dorsey AA, Osborn GG, Shebroe V, Lyles JS, et al. (2000) Evidence-based guidelines for teaching patient-centered interviewing. Patient Education and Counseling 39: 27-36.

347. Smith S, Mitchell C, Bowler S (2007) Patient-centered education: Applying learner-centered concepts to asthma education. Journal of Asthma 44: 799-804.

348. Smith-Stoner M (2011) Teaching patient-centered care during the silver hour. Online Journal of Issues in Nursing 16: 6.

349. Snyder CF, Wu AW, Miller RS, Jensen RE, Bantug ET, et al. (2011) The role of informatics in promoting patient-centered care. Cancer Journal 17: 211-218.

350. Sparks L, Villagran MM, Parker-Raley J, Cunningham CB (2007) A patient-centered approach to breaking bad news: Communication guidelines for health care providers. Journal of Applied Communication Research 35: 177-196.

351. Starfield B (2011) Is patient-centered care the same as person-focused care? Permanente Journal 15: 63-69.

352. Steiger NJ, Balog A (2010) Realizing patient-centered care: Putting patients in the center, not the middle. Frontiers of Health Services Management 26: 15-25.

353. Step MM, Rose JH, Albert JM, Cheruvu VK, Siminoff LA (2009) Modeling patient-centered communication: Oncologist relational communication and patient communication involvement in breast cancer adjuvant therapy decision-making. Patient Education and Counseling 77: 369-378.

354. Stevenson ACT (2002) Compassion and patient centred care. Australian Family Physician 31: 1103-1106.

355. Stewart M, Brown JB, Donner A, McWhinney IR, Oates J, et al. (2000) The impact of patient-centered care on outcomes. The Journal of family practice. pp. 796-804.

356. Stewart M, Brown JB, Weston WW (1989) Patient-Centred Interviewing Part III: Five Provocative Questions. Canadian Family Physician 35: 159-161.

357. Stewart M, Brown JB, Weston WW, McWhinney IR, McWilliam CL, et al. (1995) Patient-centered medicine: Transforming the clinical method. Thousand Oaks, CA: Sage Publications, Inc; US. Patient-centered medicine: Transforming the clinical method. xxiv, 267 p.

358. Stichler JF (2011) Patient-centered healthcare design. Journal of Nursing Administration 41: 503-506.

359. Stone S (2008) A retrospective evaluation of the impact of the planetree patient-centered model of care on inpatient quality outcomes. Herd 1: 55-69.

360. Stuck A, Clark MJ, Connelly CD (2011) Preventing intensive care unit delirium: A patient-centered approach to reducing sleep disruption. DCCN - Dimensions of Critical Care Nursing 30: 315-320.

361. Sturmberg JP, Reid S, Khadra MH (2002) A longitudinal, patient-centred, integrated curriculum: Facilitating community-based education in a rural clinical school. Education for Health 15: 294-304.

362. Sweeney L, Halpert A, Waranoff J (2007) Patient-centered management of complex patients can reduce costs without shortening life (Provisional abstract). American Journal of Managed Care. pp. 84-92.

363. Sweet MG (2004) A patient-centered approach to chronic disease management. JAAPA 17: 25-28.

364. Swensen SJ, Johnson CD (2010) Flying in the plane you service: Patient-centered radiology. Journal of the American College of Radiology 7: 216-221.

365. Swenson SL, Buell S, Zettler P, White M, Ruston DC, et al. (2004) Patient-centered communication: Do patients really prefer it? Journal of general internal medicine. pp. 1069-1079.

366. Swenson SL, Zettler P, Lo B (2006) 'She gave it her best shot right away': Patient experiences of biomedical and patient-centered communication. Patient Education and Counseling 61: 200-211.

367. Talley-Rostov A (2008) Patient-centered care and refractive cataract surgery. Current Opinion in Ophthalmology 19: 5-9.

368. Tandon SD, Parillo KM, Keefer M (2005) Hispanic women's perceptions of patient-centeredness during prenatal care: A mixed-method study. Birth: Issues in Perinatal Care 32: 312-317.

369. Taylor K (2009) Paternalism, participation and partnership--The evolution of patient centeredness in the consultation. Patient Education and Counseling 74: 150-155.

370. Terregino CA, Saks NS (2010) Creative group performances to assess core competencies in a first-year patient-centered medicine course. Medical Education Online 15: 4.

371. Thistlethwaite JE (1999) Introducing medical students to the concept of patient-centred consultations during a community-based teaching attachment. Medical Teacher 21: 523-526.

372. Thistlethwaite JE, Jordan JJ (1999) Patient-centred consultations: A comparison of student experience and understanding in two clinical environments. Medical Education 33: 678-685.

373. Tischler V, D'Silva K, Cheetham A, Goring M, Calton T (2010) Involving patients in research: The challenge of patient-centredness. International Journal of Social Psychiatry 56: 623-633.

374. Toop L (1998) Primary care: core values. Patient centred primary care. BMJ 316: 1882-1883.

375. Tralongo P, Ferrau F, Borsellino NV, Francesco, Caruso M, Giuffrida D, et al. (2011) Cancer patient-centered home care: A new model for health care in oncology. Therapeutics & Clinical Risk Management 7: 387-392.

376. Trojan A, Nickel S (1999) Gesundheitsfoerderung im Krankenhaus. Darstellung des europaeischen WHO-Projekts und Ergebnisse der Entwicklung und Anwendung eines Fragebogens zur Evaluation patientenorientierter Qualitaet im Krankenhaus. Health promotion in hospitals. Presentation of The European WHO project and results of the development and application of a questionnaire for the evaluation of patient-oriented quality in hospitals. Roehrle, Bernd, Sommer, Gert Praevention und Gesundheitsfoerderung Tuebingen: DGVT Deutsche Gesellschaft fuer Verhaltenstherapie (1999) Seiten 315-341 Series: Fortschritte der Gemeindepsychologie und Gesundheitsfoerderung, Band 4.

377. Tsimtsiou Z, Kerasidou O, Efstathiou N, Papaharitou SH, Konstantinos, Hatzichristou D (2007) Medical students' attitudes toward patient-centred care: A longitudinal survey. Medical Education 41: 146-153.

378. Tucker CM, Marsiske M, Rice KG, Nielson JJH, Keith (2011) Patient-centered culturally sensitive health care: Model testing and refinement. Health Psychology 30: 342-350.

379. Tufano JT, Ralston JD, Martin DP (2008) Providers' experience with an organizational redesign initiative to promote patient-centered access: A qualitative study. Journal of General Internal Medicine 23: 1778-1783.

380. Tzeng L-FC, Li-Chi, Hsueh K-C, Ma W-F, Fu L-S (2010) A preliminary study to evaluate a patient-centred asthma education programme on parental control of home environment and asthma signs and symptoms in children with moderate-to-severe asthma. Journal of Clinical Nursing 19: 1424-1433.

381. Uphoff EPMM, Wennekes L, Punt CJA, Grol RPTM, Wollersheim HCH, et al. (2012) Development of Generic Quality Indicators for Patient-Centered Cancer Care by Using a RAND Modified Delphi Method. Cancer Nursing 35: 29-37.

382. van der Eijk M, Faber MJAS, Sara, Munneke M, Bloem BR (2011) Moving towards patient-centered healthcare for patients with Parkinson's disease. Parkinsonism & Related Disorders 17: 360-364.

383. van Empel IWH, Aarts JWMC, Ben J., Huppelschoten DA, Laven JSE, Nelen WLDM, et al. (2010) Measuring patient-centredness, the neglected outcome in fertility care: A random multicentre validation study. Human Reproduction 25: 2516-2526.

384. van Empel IWH, Dancet EAF, Koolman XHE, Nelen WLDM, Stolk EA, et al. (2011) Physicians underestimate the importance of patient-centredness to patients: A discrete choice experiment in fertility care. Human Reproduction 26: 584-593.

385. van Empel IWH, Hermens RPMGA, Reinier P., Hollander KWPN, Willianne L. D. M., Kremer JAM (2011) Organizational determinants of patient-centered fertility care: A multilevel analysis. Fertility & Sterility 95: 513-519.

386. van Mossel C, Alford M, Watson H (2011) Challenges of patient-centred care: Practice or rhetoric. Nursing Inquiry 18: 278-289.

387. van Weel-Baumgarten E (2008) Patient-centered information and interventions: Tools for lifestyle change? Consequences for medical education. Family Practice 25: i67-70.

388. Vegni E, Martinoli M, Moja EA (2002) Improving patient-centred medicine: a preliminary experience for teaching communication skills to Italian general practitioners. Education for Health 15: 51-57.

389. Venetis MK, Robinson JD, Turkiewicz KLP, Allen M (2009) An evidence base for patient-centered cancer care: A meta-analysis of studies of observed communication between cancer specialists and their patients. Patient Education and Counseling 77: 379-383.

390. Wagner EH, Bennett SM, Austin BT, Greene SM, Schaefer JK, et al. (2005) Finding common ground: Patient-centeredness and evidence-based chronic illness care. The Journal of Alternative and Complementary Medicine 11: S7-15.

391. Wahlqvist M, Gunnarsson RK, Dahlgren G, Nordgren S (2010) Patient-centred attitudes among medical students: Gender and work experience in health care make a difference. Medical Teacher 32: e191-188.

392. Wanzer MB, Booth-Butterfield M, Gruber K (2004) Perceptions of health care providers' communication: Relationships between patient-centered communication and satisfaction. Health Communication 16: 363-383.

393. Wasson JH, Godfrey MM, Nelson EC, Johnson JK, Batalden PB (2007) Planning patient-centered care. Quality by design: A clinical microsystems approach. San Francisco, CA: Jossey-Bass; US. pp. 148-164.

394. Weisman E, Hagland M (1994) Built-in care. One hospital sees the future in patient-centered design. Hospitals & Health Networks 68: 54-60.

395. Wensing M, Elwyn G, Edwards A, Vingerhoets E, Grol R (2002) Deconstructing patient centred communication and uncovering shared decision making: An observational study. BMC Medical Informatics & Decision Making 2: 7.

396. West E, Barron DN, Reeves R (2005) Overcoming the barriers to patient-centred care: Time, tools and training. Journal of Clinical Nursing 14: 435-443.

397. Weston WW (2005) Patient-centered medicine: A guide to the biopsychosocial model. Families, Systems, & Health 23: 435-443.

398. Weston WW, Brown JB, Stewart MA (1989) Patient-centred interviewing part I: Understanding patients' experiences. Canadian Family Physician 35: 147-151.

399. White MA, Verhoef MJ (2005) Toward a patient-centered approach: Incorporating principles of participatory action research into clinical studies. Integrative Cancer Therapies 4: 21-24.

400. Whittemore R, Sullivan A, Bak PS (2003) Working within boundaries: A patient-centered approach to lifestyle change. Diabetes Educator 29: 69-74.

401. Wilkerson L, Fung C-C, May W, Elliott D (2010) Assessing patient-centered care: One approach to health disparities education. Journal of General Internal Medicine 25: 86-90.

402. Williams BJ (2010) The way to patient-centered care. Nursing Management 41: 10-12.

403. Williams GC, Lynch M, Glasgow RE (2007) Computer-assisted intervention improves patient-centered diabetes care by increasing autonomy support. Health psychology. pp. 728-734.

404. Williams GC, Zeldman A (2002) Patient-centered diabetes self-management education. Current Diabetes Reports 2: 145-152.

405. Winefield H, Murrell T, Clifford J, Farmer E (1996) The search for reliable and valid measures of patient-centredness. Psychology & Health 11: 811-824.

406. Wittink MN, Cary M, TenHave T, Baron J, Gallo JJ (2010) Towards patient-centered care for depression: Conjoint methods to tailor treatment based on preferences. Patient 3: 145-157.

407. Wolf D, Lehman L, Quinlin R, Rosenzweig M, Friede S, et al. (2008) Can nurses impact patient outcomes using a patient-centered care model? The Journal of nursing administration. pp. 532-540.

408. Wolf DM, Lehman L, Quinlin R, Zullo T, Hoffman L (2008) Effect of patient-centered care on patient satisfaction and quality of care. Journal of nursing care quality. pp. 3163-3121.

409. Woodcock AJ, Kinmonth A-L, Campbell MJ, Griffin SJ, Spiegal NM (1999) Diabetes care from diagnosis: Effects of training in patient-centred care on beliefs, attitudes and behaviour of primary care professionals. Patient Education and Counseling. pp. 65-79.

410. Worley-Louis MM, Schommer JC (2002) Pharmacists' therapeutic relationships with older adults: The impact of participative behavior and patient-centeredness on relationship quality and commitment. Journal of Social and Administrative Pharmacy 19: 180-189.

411. Yedidia MJ (2007) Transforming Doctor-Patient Relationships to Promote Patient-Centered Care: Lessons from Palliative Care. Journal of Pain and Symptom Management 33: 40-57.

412. Yeheskel A, Biderman A, Borkan JM, Herman J (2000) A course for teaching patient-centered medicine to family medicine residents. Academic Medicine 75: 494-497.

413. Zaleta AK, Carpenter BD (2010) Patient-centered communication during the disclosure of a dementia diagnosis. American Journal of Alzheimer's Disease & Other Dementias 25: 513-520.

414. Zandbelt LC, Smets EM, Oort FJ, de Haes HC (2005) Coding patient-centred behaviour in the medical encounter. Social Science & Medicine 61: 661-671.

415. Zandbelt LC, Smets EM, Oort FJ, Godfried MH, de Haes HC (2006) Determinants of physicians' patient-centred behaviour in the medical specialist encounter. Social Science & Medicine 63: 899-910.

416. Zandbelt LC, Smets EM, Oort FJ, Godfried MH, de Haes HC (2007) Patient participation in the medical specialist encounter: Does physicians' patient-centred communication matter? Patient Education and Counseling 65: 396-406.

417. Zandbelt LC, Smets EM, Oort FJ, Godfried MH, de Haes HC (2007) Medical specialists' patient-centered communication and patient-reported outcomes. Medical Care 45: 330-339.
